# Comparison of the effects of different physical stimulation therapies on reducing upper limb spastic paralysis and motor dysfunction in stroke survivors after stroke: a network meta-analysis of randomized controlled trials

**DOI:** 10.3389/fneur.2025.1554583

**Published:** 2025-04-15

**Authors:** Mingtong Bian, Fuyan Chen, Huizhen Su, Zhiying Li, Xiaowei Sun, Yang Liu, Jinyuan Shi, Shuo Liu, Ru Rong

**Affiliations:** ^1^Department of Acupuncture, First Teaching Hospital of Tianjin University of Traditional Chinese Medicine, Tianjin, China; ^2^National Clinical Research Center for Chinese Medicine Acupuncture and Moxibustion, Tianjin, China; ^3^Qinghai Provincial Hospital of Traditional Chinese Medicine, Xining, Qinghai, China

**Keywords:** spasticity, stroke, rehabilitation, review, network meta-analysis

## Abstract

**Background:**

Upper limb spasticity is a common and disabling sequela of stroke, which significantly impairing motor function and the capacity to perform activities of daily living (ADL). The relative efficacy of different physical therapies and their combinations compared to monotherapies remains unclear.

**Methods:**

A comprehensive database search was conducted to identify randomized controlled trials (RCTs) published from database inception to 2024 that evaluated physical therapies for post-stroke upper limb spasticity. Data were analyzed using RevMan and STATA/R software with a Bayesian framework for network meta-analysis. Evidence consistency was assessed via node-splitting approaches, and intervention efficacy was ranked using the surface under the cumulative ranking curve (SUCRA). Effect sizes were expressed as mean differences (MD) with 95% confidence intervals (CI), and study quality was evaluated using the Grading of Recommendations, Assessment, Development, and Evaluations (GRADE) system.

**Results:**

Forty-nine RCTs involving 3,219 patients were included. The combination of physical rehabilitation (PR) with repetitive transcranial magnetic stimulation (rTMS) and electro-acupuncture (EA) demonstrated the highest improvement in Fugl-Meyer Assessment for Upper Extremity (FMA-UE) scores (91.1%), outperforming PR alone (13.2%) or EA monotherapy (30.3%). PR combined with rTMS and body acupuncture (BA) shows the most significant improvement in the Modified Barthel Index (MBI) (83.1%), superior to PR (20.8%) or BA (23.8%) alone. Adverse events (e.g., minor bruising from EA) were infrequent and self-resolving.

**Conclusion:**

Current evidence indicates that synergistic application of PR with rTMS and acupuncture (EA/BA) significantly enhances upper limb motor function and ADL capacity. However, GRADE evaluations rated most evidence as moderate quality, limited by implementation bias, insufficient subgroup analyses, and lack of long-term follow-up data. Future studies should adopt standardized protocols and investigate efficacy variations across stroke subtypes.

**Systematic review registration:**

https://www.crd.york.ac.uk/PROSPERO/view/CRD42025633289, identifier [CRD42025633289].

## 1 Introduction

Upper limb dysfunction following a stroke represents a significant cause of long-term disability in patients, frequently occurring in conjunction with a range of injuries, including upper limb weakness and spasticity. The principal manifestation of upper limb spastic paralysis is an increase in muscle tone on the affected side, which is characterized by symptoms such as shoulder adduction and internal rotation, elbow flexion and pronation, wrist flexion and ulnar deviation, and finger clenching (1). This can result in a number of adverse effects, including pain, muscle contraction, changes in soft tissue structure, weakness, associated reactions, loss of passive function, limited active function, and a decrease in quality of life. This has a significant impact on the patient’s activities of daily living (2). The pathological mechanism of upper limb spasticity is complex, involving damage to the corticospinal tract, peripheral mechanisms, extensor mechanisms, and potential spastic dystonia, among other factors (3). Furthermore, because the upper limb’s role in more refined and diverse functions, the recovery of its dysfunction is more complex and slow, posing significant challenges to the patient’s daily life and social participation.

Nevertheless, research has demonstrated that spasticity can be effectively managed in the chronic phase of stroke through appropriate intervention, thereby enhancing motor function and facilitating the restoration of limb function (4). It is therefore imperative to identify and investigate efficacious rehabilitation techniques to facilitate enhanced recovery of upper limb function in patients. At present, there is a general consensus on the rehabilitation treatment for this condition, both domestically and internationally. The aforementioned treatments are primarily comprised of physical exercise and occupational therapy. In recent years, the advancement of medical technology and the intensification of clinical research have given rise to a multitude of novel rehabilitation therapies, including acupuncture, massage, proprioceptive neuromuscular facilitation (PNF), repetitive transcranial magnetic stimulation (rTMS), and theta-burst stimulation (TBS). A number of studies (5–8) have demonstrated that these physical therapies can facilitate the improvement of post-stroke spastic paralysis to a certain extent. However, existing research has predominantly focused on monotherapies, with insufficient comparative investigations of multimodal therapeutic regimens, leaving the optimal therapeutic combinations poorly defined.

Network meta-analysis (NMA) overcomes the limitations of traditional pairwise meta-analyses, which are restricted to comparing two interventions at a time, by integrating direct and indirect evidence to systematically evaluate the synergistic effects of complex multimodal rehabilitation strategies within a unified framework (9). This study applied NMA to compare the efficacy of 21 intervention modalities for post-stroke upper limb spasticity, aiming to provide evidence-based insights for personalized, multimodal rehabilitation protocols. A total of 49 randomized controlled trials (RCTs) involving 3,219 participants, published between 2009 and 2024, were included. Outcomes were quantified using the Fugl-Meyer Assessment for Upper Extremity (FMA-UE) for motor function and the Modified Barthel Index (MBI) for activities of daily living (ADL). A Bayesian network meta-analysis (implemented in STATA/R) was conducted to comprehensively assess efficacy differences among rehabilitation therapies, neuromodulation techniques, and integrative traditional Chinese medicine (TCM) regimens. Interventions were ranked via the surface under the cumulative ranking curve (SUCRA). The results elucidated effectiveness hierarchies through probabilistic estimates and established indirect efficacy comparison pathways for interventions lacking direct comparative data. This framework provides clinicians and patients with a scientific foundation for optimizing combined strategies of rehabilitation, neuromodulation, and TCM therapies, while bridging critical evidence gaps in the current literature on post-stroke spasticity management.

## 2 Methods

### 2.1 Registration

The evaluation plan of this system has been registered with the International Prospective Register of Systematic Reviews (PROSPERO) under registration number CRD42024607022. This study was conducted in accordance with the Preferred Reporting Items for Systematic Reviews and Meta-Analyses Extension for Network Meta-Analyses (PRISMA-NMA), as detailed in the Supplementary Appendix S1.

### 2.2 Search strategy

A systematic search of the following databases was conducted in order to identify eligible randomized controlled trials (RCTs): The following databases were searched: PubMed, Embase, Cochrane Library, Web of Science, China National Knowledge Infrastructure (CNKI), China Biomedical Literature Database (CBM), China Science and Technology Journal (VIP) database, and Wanfang Database. The search was conducted from the inception of the databases to October 2024, with the search terms limited to Chinese or English language sources. Furthermore, the reference lists of the retrieved relevant review articles were examined to ascertain whether any additional literature had been overlooked. The search strategy employed the following keywords: (“stroke” OR “cerebrovascular accident” OR “cerebral infarction” OR “cerebral haemorrhage”) AND (“spastic paralysis” OR “rigid paralysis” OR “paralysis, spastic”) AND (“upper extremity”) AND (“acupuncture” OR “massage” OR “rTMS” OR “low-frequency electrical stimulation”). Additionally, the search was conducted in Chinese databases using Chinese characters with the same meanings (shown in Supplementary Appendix S2).

### 2.3 Literature selection criteria

The literature screening and adjustment were conducted in accordance with the inclusion and exclusion criteria set forth in Table 1.

**Table 1:** Eligibility criteria for relevant studies.

Criteria	Inclusion	Exclusion
Population	Patients diagnosed with a stroke through head CT or MRI scans, exhibiting increased muscle tone, brisk tendon reflexes, and the presence of pathological reflexes on the affected side of the upper limb, are classified as having negative or positive pathological reflexes. Eligible study participants were adults over 18 years of age, with no limitations regarding gender or disease duration.	Spastic paralysis due to etiologies such as head trauma. Under the age of 18 years
Intervention	The physical therapy programme encompasses individual and combined treatments, including acupuncture (electroacupuncture and body acupuncture), massage, PNF techniques, ESWT, rTMS, cTBS, iTBS, and physical rehabilitation.	Other non-pharmacological treatments not covered by the study
Comparators	Physical rehabilitation (Traditional rehabilitation therapy without the use of mechanical aids, such as manual physical therapy and traditional exercise therapy)	Other non-pharmacological treatments not covered by the study
Outcomes	Fugl-Meyer Assessment-Upper Extremity (FMA-UE) Modified Barthel Index (MBI) Adverse events (AEs)	Lack of valid outcome
Languages	Chinese and English	Other languages
Study designs	Randomized controlled trials (RCTs)	Non-randomized controlled trials (RCTs) and conference papers

### 2.4 Data collection and extraction

Two researchers (JY-S and S-L) conducted the preliminary search and excluded titles and abstracts that were not pertinent to the subject matter of this review, while also cross-verifying the screening results. Furthermore, two additional researchers (MT-B and XW-S) conducted independent evaluations of the remaining titles and abstracts, obtained the full texts of these studies, and determined whether they met the inclusion criteria. They also cross-checked these results. Only after confirming that the full-text literature met the inclusion criteria was it included in the study, and the relevant Data were analyzed were then extracted. The extracted content comprised the following elements: basic study information (first author, publication year, diagnostic criteria, number of participants), study design (including sample size, specific description of interventions, type of control group, duration of treatment, treatment cycle, frequency), participant characteristics (age, gender, type of stroke and duration of stroke), outcomes, and data on the quality of the studies (randomization method, allocation concealment, implementation of blinding, loss to follow-up or withdrawal, etc.). Subsequently, an additional researcher (FY-C) undertook an independent review of the extracted data. Any discrepancies were resolved through discussion with FY-C.

### 2.5 Quality assessment

The methodological quality of each study was evaluated by two researchers (MT-B and XW-S) using the Cochrane Risk of Bias tool (ROB2). The Cochrane tool identifies seven potential areas of bias, including sequence generation, allocation concealment, blinding of participants and personnel, incomplete outcome data, selective outcome reporting, and other biases. The risk of bias and quality of evidence for each domain can be categorized as low risk, unclear risk (insufficient detail or not reported), or high risk of bias. In order to assess the quality of the included literature, the Consolidated Standards of Reporting Trials (CONSORT) guidelines (10) were adopted. Furthermore, the quality of evidence for each outcome measure was evaluated using the Grading of Recommendations Assessment, Development, and Evaluation (GRADE) system (11), with ratings classified as high, moderate, low, or very low levels, respectively. Any discrepancies that arose during the assessment process were resolved by a third researcher (FY-C).

### 2.6 Statistical analysis

Pairwise meta-analyses were conducted utilizing RevMan 5.4 software (Cochrane Collaboration, Oxford, United Kingdom). In the case of continuous data, the mean difference (MD) and its 95% confidence interval (CI) were employed as a means of measuring the effect size. In the case of binary data, the effect size was evaluated using the odds ratio (OR) and its 95% confidence intervals (CIs). The extent of heterogeneity among the included studies was assessed using Cochran’s Q test (*p*-value) and Higgins’s I^2^ statistic. If *p* ≥ 0.05 and I^2^ ≤ 50%, heterogeneity was considered acceptable, and a fixed-effect model was used. Otherwise, a random-effects model was selected.

In conducting the network meta-analysis, the STATA 14.0 (Stata Corp, College Station, Texas, United States) and the R 4.3.3 (maintained by the R Core Team, Vienna, Austria) were employed to perform the requisite analysis within a Bayesian framework. In the event of a closed loop of evidence, an initial assessment of the inconsistency of the evidence was conducted. An inconsistency model was constructed to ascertain whether the *p*-value exceeded 0.05. If the *p*-value is greater than 0.05, this indicates that there is no significant inconsistency and that a consistency model should be selected for subsequent effect size estimation. Conversely, if the *p*-value was less than or equal to 0.05, this indicated significant inconsistency across the studies. In such cases, it was necessary to investigate the sources of inconsistency and consider the use of an inconsistency model or the implementation of sensitivity analyses to assess the potential impact of this inconsistency on the study results. In view of the potential heterogeneity of the included studies, a random-effects model was employed for the synthesis of the data. As the outcome variables of the studies were continuous, the effect size was measured using mean differences (MDs) and 95% confidence intervals (CIs). Markov Chain Monte Carlo (MCMC) methods were employed to estimate the model, with four chains configured, 20,000 iterations, and a burn-in period of 5,000, setting a thinning interval of 1. To confirm model convergence, Brooks-Gelman-Rubin diagnostics plots, chain trace plots, and probability density plots were examined. The node-splitting method was employed to assess the consistency of direct and indirect comparisons. If the resulting *p*-value was greater than 0.05, it was inferred that there was a higher degree of consistency. In instances where closed-loop comparisons were present, the inconsistency factor (IF) was utilized for evaluation purposes. If the 95% CI encompassed 0, this indicated that there was consistency between the direct and indirect evidence. Furthermore, the Surface Under the Cumulative Ranking (SUCRA) was calculated to probabilistically rank the various treatment interventions, with SUCRA scores ranging from 0 to 100%, where higher scores indicated superior treatment effectiveness. In analyzing the result data, consideration was given to the potential impact of baseline differences by employing a correlation coefficient R value of 0.5 in the following formula (Equations 1, 2) for estimation.


(1) $${\bar{MDs}}_{Change}={\bar{MDs}}_{Final}-{\bar{MDs}}_{Baseline}$$

(2) $$\begin{array}{c} \begin{array}{l} SD_{Change}=\\ \sqrt{{\left(SD_{Baseline}\right)}^{2}+{\left(SD_{Final}\right)}^{2}-\left(2\times R\times SD_{Baseline}\times SD_{Final}\right)} \end{array} \end{array}$$

## 3 Results

### 3.1 Results of the search

**Figure 1:**
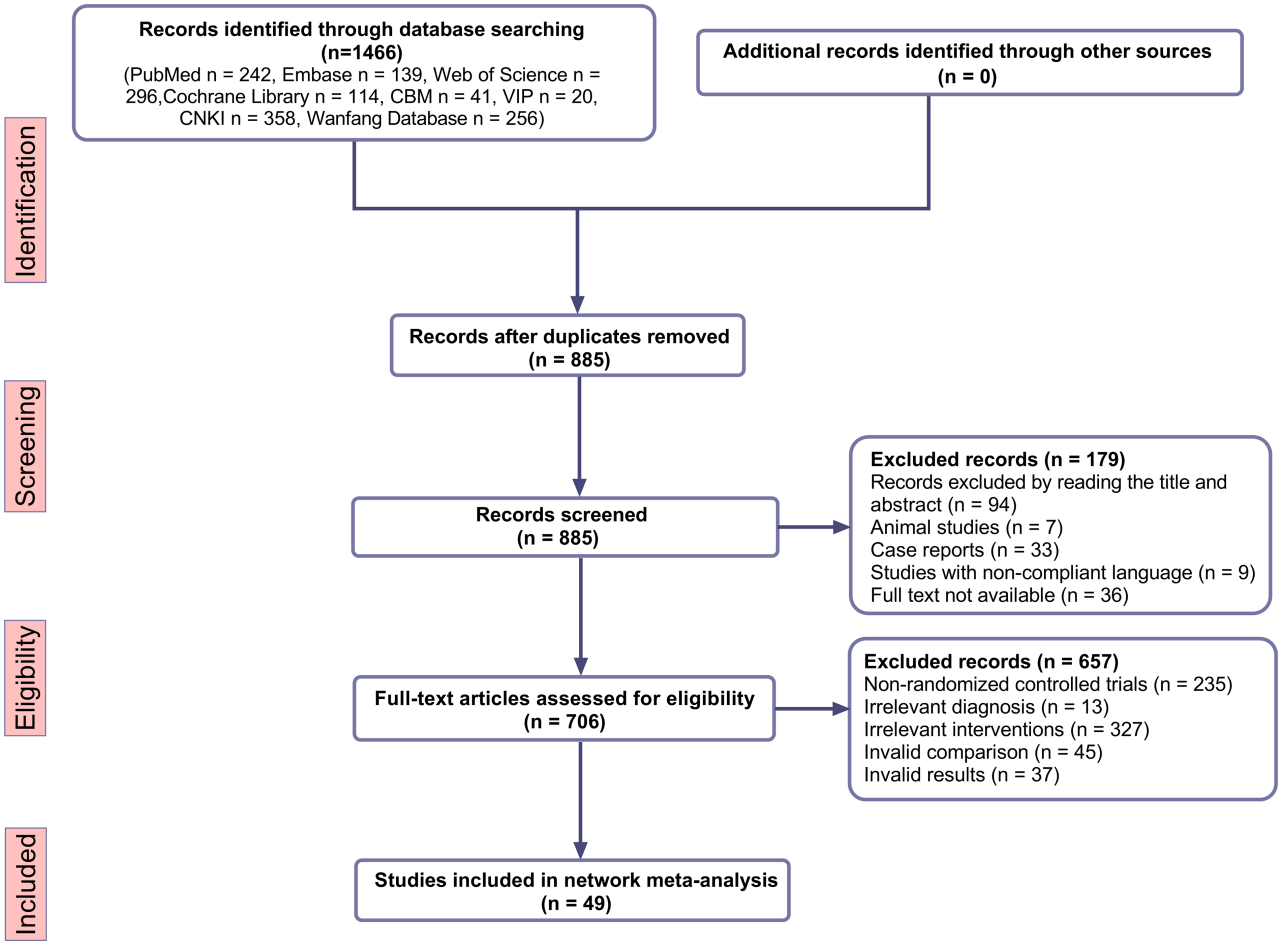
Flowchart of literature screening.

In accordance with the established inclusion criteria, our preliminary search yielded a total of 1,466 published studies. Following the preliminary review, 581 studies were identified as duplicates and subsequently removed. The remaining 885 studies were then subjected to independent examination by two researchers, with their titles and abstracts analyzed. A total of 179 studies were excluded on the basis of their irrelevance to the research question. Subsequently, a comprehensive review was conducted on the 706 selected studies, with a detailed assessment of their study design, participant population, interventions, and outcome measurements. In conclusion, a total of 49 RCTs (12–60) were included in the final analysis. The process of study selection is outlined in detail in the PRISMA flow diagram (shown in Figure 1).

The studies included in the analysis spanned a period of approximately 15 years, from September 2009 to March 2024. The studies were distributed across a number of countries, including mainland China (*n* = 44), Taiwan, China (*n* = 2), Brazil (*n* = 1), Turkey (*n* = 1) and Iran (*n* = 1) Of the included trials, 42 RCTs (12, 13, 15–28, 30–35, 37, 38, 41, 42, 44–55, 57–60) were two-arm designs (85.71%), 6 RCTs (14, 29, 36, 39, 40, 56) were three-arm designs (12.24%), and 1 RCTs (43) was a four-arm experiment (2.04%). The studies exhibited considerable variation in terms of sample size, duration of treatment, and intervention measures. In total, the studies recruited 3,219 participants, with 1,697 allocated to the experimental group and 1,522 to the control group. The number of participants ranged from 12 (19) to 204 (45). The baseline characteristics of the participants in the two groups were generally similar, with the average age being 60.24 years (standard deviation 9.15). However, one study (16) did not provide data on the mean age. Seven studies (15, 22, 30, 36, 43, 44, 58) did not report the mean duration of disease. Among the remaining 42 studies, the mean duration of disease ranged from (8.2 ± 6.6) days (27) to (58.9 ± 27.2) months (18). Regarding disease phases, the majority of studies (12–14, 16, 17, 20, 21, 23–26, 28, 29, 31, 33–35, 37, 38, 40–42, 45, 46, 48–57, 59, 60) (75.5%, 37/49) involved patients in the subacute phase, whereas only 4 (18, 19, 32, 39) and 1 studies (27) focused on chronic and acute phases, respectively. With the exception of one study (19) that did not provide gender information, the proportion of male participants among those who had experienced a stroke was 59.34%. Four studies (23, 29, 38, 58) provided data on patient dropout and the specific reasons for this, with the number of dropouts ranging from one to five individuals.

The included RCTs employed seven physical rehabilitation treatment methods, including physical rehabilitation (PR, encompassing exercise training and functional activity training, among others). The remaining treatments were acupuncture therapy (including body acupuncture BA and electro-acupuncture, EA), massage (M), PNF, rTMS, extracorporeal shock wave therapy (ESWT), and TBS (including continuous theta burst stimulation cTBS and intermittent theta burst stimulation iTBS). These physical rehabilitation treatments may be applied either individually or in combination, forming a total of 21 distinct treatment strategies. The core operational parameters of the interventions exhibited limited overall heterogeneity (shown in Supplementary Table S1), with the following modality-specific patterns: In the 16 studies (18–20, 25, 28, 31, 37, 39, 40, 44, 48, 52, 54, 57, 58, 60) that employed rTMS, a low-frequency stimulation pattern of 1 Hz was frequently utilized. However, there was some variation in stimulation intensity thresholds, ranging from 60 to 120% of resting motor threshold (RMT) or active motor threshold (AMT). Furthermore, the target of stimulation in all studies focused on the primary motor cortex (contralesional M1) contralateral to the lesion. Among the 6 studies (43, 46, 47, 55, 56, 59) employing ESWT, five (46, 47, 55, 56, 59) opted for a frequency of 8 Hz, while one (43) selected 5 Hz. The energy intensity was modulated based on anatomical location, with the majority of upper limb treatment parameters ranging from 1.0 to 3.0 bar. For instance, Ai et al. (56) utilized a gradient strategy (1.0–2.0 bar for the upper limb and 2.0–3.0 bar for the elbow-shoulder complex) depending on the site, whereas Chen et al. (59) employed a uniform intensity program (3.0 bar). Of the 4 studies (20, 30, 38, 40) employing TBS, all utilized 80% AMT. In acupuncture treatments, the duration of a single stimulation of BA ranged from 15–30 min, and all studies (13, 16, 21, 23, 24, 27, 29, 32–35, 41–43, 46, 47, 50, 51, 53, 55, 56, 58, 60) using BA focused on upper limb acupoints. The frequency parameters of EA exhibited a bimodal distribution, with a low-frequency group (2–5 Hz) (14, 52) and a high-frequency group (50–100 Hz) (12). Among the 8 studies (15, 22, 26, 35, 36, 41, 45, 49) employing M therapy, the single-session intervention duration ranged from 10 to 40 min, with all protocols exclusively applied to the spastic upper limb.

In terms of outcomes, a total of 41 studies (14, 15, 18, 24, 26, 27, 30, 37, 39, 49, 52, 53, 56, 59–61) employed the Fugl-Meyer Assessment-Upper Extremity (FMA-UE) scale, a tool with a maximum score of 66 points, designed to assess patients’ motor function, balance, joint pain, and range of motion. Furthermore, 34 studies (14, 19, 21–23, 25–28, 31, 32, 34–38, 40–44, 46–49, 51–53, 56–61) employed the Modified Barthel Index (MBI) scale to assess patients’ abilities in activities of daily living. The MBI has a total score of 100 points and encompasses aspects such as self-care ability, mobility, and degree of dependence. In both assessment tools, a higher score indicates superior functional performance of the patient. Additional details regarding the characteristics of the studies are presented in Table 2.

**Table 2:** Characteristics of the included studies.

Author	Year	Sample (T/C) (M/F)	Age (year)	Stroke type (I/H)	Course of disease	Treatment	Intervention period	Outcome	Drop-out situation (T/C)
Ai YX	2023	19/11	55 ± 4	-	(4.56 ± 0.89) w	PR+ESWT+BA	4 w	FMA-UE MBI	None
		17/13	56 ± 5	-	(4.23 ± 0.93) w	PR+BA			
		14/16	57 ± 4	-	(4.16 ± 0.78) w	PR			
Bao YH	2012	23/23	67.39 ± 9.75	34/12	(2.63 ± 1.42) m	PR+EA	4 w	FMA-UE	None
		19/22	66.56 ± 9.65	32/9	(2.58 ± 1.46) m	EA			
		22/20	64.85 ± 8.90	31/11	(2.59 ± 1.41) m	PR			
Barros G	2014	6/4	57.4 ± 12.0	9/1	(47.8 ± 43.2) m	PR+rTMS	4 w	FMA-UE	None
		7/3	64.6 ± 6.8	8/2	(58.9 ± 27.2) m	PR			
Chen DY	2024	17/13	68.74 ± 5.23	18/12	(26.41 ± 4.29) d	PR+ESWT	4 w	FMA-UE MBI	None
		18/12	69.11 ± 6.14	20/10	(27.31 ± 5.64) d	PR			
Chen QF	2021	20/10	64.13 ± 13.20	26/4	(2.00 ± 1.34) m	PR+rTMS	4 w	FMA-UE MBI	None
		20/10	61.37 ± 11.90	23/7	(2.17 ± 11.1) m	PR			
Chen Y	2021	13/3	57.38 ± 8.04	10/6	(80.13 ± 35.19) d	PR+iTBS	2 w	MBI	2
		12/4	51.44 ± 9.19	8/8	(101.50 ± 54.15) d	PR			0
Chen YJ	2019	7/4	52.9 ± 11.1	2/9	-	PR+iTBS	2 w	FMA-UE	None
		7/4	52.6 ± 8.3	3/8	-	PR			
Chu GX	2009	18/12	60.37 ± 10.81	25/5	(42.33 ± 16.72) d	PR+EA	4 w	MBI	None
		16/14	60.77 ± 10.65	24/6	(40.20 ± 14.06) d	PR			
Dang YS	2020	20/15	55.23 ± 8.48	18/17	(50.28 ± 16.32) d	PR+BA	4 w	MBI	None
		19/16	55.26 ± 8.51	20/15	(50.24 ± 16.27) d	PR			
Gu YL	2018	26/14	58.01 ± 10.14	-	(48.50 ± 12.12) d	PR+M	3 w	FMA-UE MBI	None
		25/15	56.12 ± 11.06	-	(50.92 ± 12.03) d	PR			
Hao JB	2016	26/14	61.30 ± 9.33	28/12	-	PR+M	4 w	MBI	None
		24/16	60.96 ± 8.76	26/14	-	PR			
Jiang YY	2023	13/12	56.72 ± 10.50	-	(2.62 ± 1.18) m	PR+rTMS+EA	4 w	FMA-UE MBI	None
		17/8	54.56 ± 12.68	-	(2.66 ± 1.12) m	PR+EA			
Kuzu Ö	2021	4/3	56.3 ± 11.5	7/0	(16.4 ± 2.5) m	PR+rTMS	10 w	FMA-UE	None
		6/1	61.3 ± 9.8	7/0	(14.5 ± 1.6) m	PR+cTBS			
		4/2	65.0 ± 4.6	6/0	(14.5 ± 2.0) m	PR			
Lei JF	2024	18/2	58.90 ± 9.49	16/4	(1.82 ± 1.25) m	PR+rTMS+BA	4 w	FMA-UE MBI	None
		11/9	59.10 ± 11.92	12/8	(1.18 ± 0.63) m	PR+BA			
Lei M	2012	27/19	64.91 ± 8.85	31/15	-	PR+M	12 w	FMA-UE	None
		27/14	63.66 ± 9.02	26/15	-	PR			
Li B	2021	22/13	62.53 ± 2.75	-	(45.86 ± 1.54) d	BA+M	8 w	MBI	None
		19/16	62.46 ± 2.87	-	(45.93 ± 1.65) d	BA			
Li BJ	2017	32/28	55.7 ± 4.8	-	(15.2 ± 3.7) d	PR+BA	8 w	FMA-UE	None
		35/25	54.9 ± 5.2	-	(15.6 ± 3.3) d	PR			
Li D	2021	20/10	56.77 ± 8.58	24/6	(3.63 ± 1.85) m	PR+rTMS+cTBS	4 w	MBI	None
		19/11	57.60 ± 7.40	23/7	(3.80 ± 1.71) m	PR+rTMS			
		18/12	55.13 ± 7.90	24/6	(3.67 ± 1.84) m	PR+cTBS			
Li ZW	2022	18/12	60.27 ± 6.14	16/14	(8.97 ± 4.14) w	PR+M	4 w	FMA-UE MBI	None
		16/14	59.93 ± 7.15	19/11	(9.30 ± 4.55) w	PR			
Lin FY	2018	17/13	59 ± 5	25/5	(8.2 ± 6.6) d	PR+BA	4 w	FMA-UE MBI	None
		16/14	59 ± 7	26/4	(9.3 ± 6.3) d	PR			
Liu HJ	2023	18/12	55.23 ± 7.86	25/5	(3.83 ± 1.03) m	PR+BA	4 w	FMA-UE MBI	0
		17/12	54.83 ± 13.92	20/9	(3.99 ± 0.96) m	PR			1
Liu QQ	2021	19/12	55.51 ± 3.20	-	(45.78 ± 5.45) d	BA+PNF	4 w	FMA-UE MBI	None
		18/13	55.42 ± 3.19	-	(45.21 ± 5.23) d	PNF			
Liu SD	2023	28/22	73.05 ± 6.31	-	(21.41 ± 5.61) d	EA+rTMS	4 w	FMA-UE	None
		30/20	72.37 ± 5.63	-	(20.21 ± 5.44) d	EA			
Liu SH	2019	7/13	61.35 ± 9.43	-	(2.81 ± 1.27) m	PR+rTMS	4 w	FMA-UE MBI	None
		11/9	55.00 ± 11.86	-	(3.11 ± 1.37) m	PR			
Liu Y	2018	5/5	56.90 ± 9.02	-	(4.50 ± 1.90) m	PR+rTMS	8 w	FMA-UE MBI	None
		9/4	55.38 ± 8.40	-	(4.85 ± 2.08) m	PR			
Ma AF	2022	30/12	61 ± 6	32/10	(27.8 ± 3.8) d	PR++BA	4 w	FMA-UE	None
		32/10	60 ± 6	31/11	(27.3 ± 3.6) d	PR			
Ma JY	2020	17/13	60.47 ± 3.98	-	(49.13 ± 4.48) d	BA+M	8 w	MBI	None
		19/11	60.43 ± 3.73	-	(48.60 ± 2.88) d	BA			
Motamed V	2014	6	55.17 ± 5.42	-	(24.00 ± 8.29) m	PR+rTMS	3 w	FMA-UE MBI	None
		6	57.00 ± 8.67	-	(23.00 ± 8.94) m	PR			
Ni HH	2012	36/14	-	34/16	(35.02 ± 6.82) d	PR+BA	4 w	FMA-UE	None
		34/16	-	33/17	(35.20 ± 6.40) d	PR			
Qin Y	2023	9/6	55.87 ± 10.50	-	(3.20 ± 1.93) m	PR+rTMS	8 w	FMA-UE MBI	None
		11/3	59.43 ± 9.12	-	(2.85 ± 1.74) m	PR			
Shi J	2019	12/8	58.5 ± 9.5	-	(67.3 ± 45.9) d	BA+PNF	4 w	FMA-UE MBI	None
		13/7	55.2 ± 13.9	-	(72.3 ± 48.6) d	BA			
Sun X	2023	16/14	55.83 ± 11.05	-	(29.60 ± 6.48) d	PR+ESWT+BA	4 w	FMA-UE	None
		17/13	58.30 ± 10.95	-	(29.13 ± 5.50) d	PR+BA			
Sun YZ	2013	17/13	62.82 ± 7.93	-	(55.44 ± 8.30) d	PR+EA	4 w	FMA-UE	None
		15/15	63.50 ± 6.51	-	(59.67 ± 6.81) d	PR			
Tong JY	2022	32/26	60.62 ± 5.63	-	(58.35 ± 6.21) d	PR+BA	4 w	FMA-UE MBI	None
		31/27	60.45 ± 5.59	-	(58.27 ± 6.17) d	PR			
Wang CP	2014	14/3	62.2 ± 12	-	(4.6 ± 3.9) m	PR+rTMS+iTBS	4 w	FMA-UE	None
		11/5	62.5 ± 13.4	-	(4.4 ± 3.1) m	PR			
Wang J	2018	15/15	53.75 ± 7.97	18/12	(50.43 ± 16.93) d	PR+BA	4 w	FMA-UE	1
		17/13	55.17 ± 8.46	19/11	(50.26 ± 16.34) d	BA			1
		16/15	54.91 ± 8.76	17/14	(54.91 ± 8.76) d	PR			1
Wei CB	2021	10/10	56.7 ± 10.5	20/0	-	PR+ESWT+BA	4 w	FMA-UE MBI	None
		11/9	57.5 ± 9.4	20/0	-	PR+ESWT			
		13/7	56.3 ± 11.4	20/0	-	PR+BA			
		12/8	55.3 ± 10.4	20/0	-	PR			
Wen DG	2020	12/8	57.15 ± 11.04	-	-	PR+M	3 w	FMA-UE MBI	None
		14/6	62.60 ± 8.99	-	-	M			
		14/4	62.15 ± 8.97	-	-	PR			
Xie WX	2023	13/6	58.42 ± 12.76	14/5	-	PR+rTMS+BA	2 w	MBI	1
		10/7	54.47 ± 9.152	10/7	-	PR+BA			3
Xu SF	2016	28/8	60 ± 10	-	(50.39 ± 22.52) d	PR+BA	4 w	FMA-UE MBI	2
		24/11	65 ± 6	-	(47.75 ± 22.63) d	PR			3
Xu YL	2010	17/15	57 ± 7.3	17/15	(48.73 ± 19.52) d	BA	12 w	FMA-UE	None
		14/17	58 ± 4.7	16/15	(52.49 ± 21.65) d	PR			
Yang NY	2017	12/8	60.7 ± 12.2	16/4	(37.5 ± 26) d	PR+rTMS	2 w	FMA-UE MBI	None
		17/3	58.7 ± 12.7	13/7	(42.5 ± 30.6) d	PR			
Yang X	2021	8/6	60.86 ± 12.396	-	-	PR+rTMS	8 w	FMA-UE MBI	None
		4/7	66.09 ± 7.436	-	-	PR			
Zhang L	2015	22/18	51.6 ± 10.4	31/9	(2.7 ± 1.2) m	PR+BA	8 w	FMA-UE MBI	None
		24/16	52.1 ± 8.6	33/7	(2.5 ± 1.3) m	PR			
Zhang QF	2021	48/54	53.47 ± 3.81	-	(2.34 ± 0.75) m	PR+M	4 w	FMA-UE	None
		58/44	53.84 ± 3.29	-	(2.15 ± 0.63) m	PR			
Zhang X	2021	18/17	50.66 ± 8.77	29/6	(2.43 ± 1.32) m	PR+ESWT+BA	4 w	FMA-UE MBI	None
		19/16	52.63 ± 8.64	29/6	(2.31 ± 1.56) m	PR+BA			
Zhao J	2021	36/14	56.32 ± 7.83	-	(2.87 ± 0.82) m	PR+rTMS	4 w	FMA-UE MBI	None
		35/15	56.29 ± 7.88	-	(2.81 ± 0.79) m	PR			
Zhao JY	2021	18/12	66.1 ± 1.6	-	(29.8 ± 1.5) d	BA+ ESWT	3 w	FMA-UE MBI	None
		16/14	67.8 ± 1.8	-	(30.2 ± 2.0) d	BA			
Zhou P	2019	17/13	60 ± 9	12/18	(45.4 ± 21.1) d	PR+BA	4 w	FMA-UE MBI	None
		16/14	61 ± 8	14/16	(44.1 ± 20.2) d	BA			

T, Treatment group; C, Control group; M, Man; F, Female; I, Ischemic Stroke; H, Hemorrhagic stroke; PR, Physical rehabilitation; BA, Body acupuncture; EA, Electro-acupuncture; M, Massage; PNT, Proprioceptive Neuromuscular Facilitation; ESWT, Extracorporeal shock wave treatment; rTMS, Repetitive transcranial magnetic stimulation; iTBS, Intermittent theta burst stimulation; cTBS, Continuous theta burst stimulation; FMA-UE, The Fugl-Meyer Assessment-Upper Extremity scale; MBI, The Modified Barthel Index scale.

Follow-up outcomes were reported in 7 studies (18, 20, 25, 39, 46, 48, 54), revealing time-dependent therapeutic effects: A study (48) conducted post-intervention revealed that patients in the low-frequency rTMS group exhibited a significantly higher MBI scores at the 2-week follow-up when compared to the conventional group (*p* < 0.05), thereby suggesting an early effect of enhanced ability in performing ADL. Three studies found that at 4-week follow-up, myotonia modified Ashworth scale (MAS) scores were reduced by ≥1 in the rTMS group by up to 55.5% (18) and that combined cTBS maintained upper limb motor function and improved carpal flexor spasticity (39), but did not significantly enhance ADL independence (25). Three-month follow-up data suggest that combined rTMS with an iTBS regimen resulted in sustained improvements in motor function (20), with a significantly lower relapse rate in the observation group than in the control group (54). Furthermore, Zhang et al. (46) reported a significant improvement in self-assessed outcome (PRO) scores from baseline in both groups (*p* < 0.05).

### 3.2 Risk-of-bias assessment

The results of the bias risk assessment for the included studies are presented in Supplementary Figure S1. Three studies (23, 29, 56, 58) were classified as exhibiting a high risk of bias, six (18, 25, 30, 34, 39, 43) were deemed to have a low risk of bias, and the remaining studies were situated between these two categories, indicating a certain level of bias risk. While the majority of studies adhered to the fundamental tenets of the CONSORT statement, the absence of certain essential information is a notable shortcoming. For example, deficiencies were identified in the description of intervention similarity, discussion of trial limitations, and assessment of external validity. The reporting of blindness and allocation concealment, two fundamental methods for controlling bias, was inadequate, thereby further undermining the reliability of the trial results. Furthermore, the majority of studies did not indicate whether they had been registered, which restricts the capacity to evaluate the transparency and reliability of the trials. Further detailed assessment information can be found in Supplementary Appendix S3.

### 3.3 Pairwise meta-analysis

In order to evaluate the impact of different interventions on the improvement of patients’ upper limb function, a comprehensive analysis was conducted on studies utilizing the same treatment and observing the same outcome indicators. This analysis was employed to facilitate direct paired meta-analyses for the FMA-UE and MBI, with 25 and 22 studies, respectively. For the FMA-UE scores, the following interventions were compared to PR: BA (two RCTs; MD = 5.6, 95% CI: 0.90, 10, p = 0.39), PR+rTMS (ten RCTs; MD = 7.2, 95% CI: 4.4, 9.9, p < 0.00001), PR+ESWT (two RCTs; MD = 7.2, 95% CI: 2.4, 12, p < 0.00001), PR+EA (two RCTs; MD = 12, 95% CI: 5.6, 18, p < 0.00001), PR+BA (eleven RCTs; MD = 6.1, 95% CI: 4.0, 8.2, p = 0.68), PR+M (five RCTs; MD = 7.2, 95% CI: 3.9, 11, p < 0.00001), PR+ESWT+BA (five RCTs; MD = 7.5, 95% CI: 2.8, 12, p = 0.15), all showing superior effects to PR. BA+ESWT (one RCT; MD = 11, 95% CI: 3.1, 19, p < 0.00001) was more effective than BA alone, EA+rTMS (one RCT; MD = 8.3, 95% CI: 1.0, 16, p < 0.00001) was more effective than EA alone, PR+M (one RCT; MD = 16, 95% CI: 5.1, 27, p < 0.00001) had a better effect than M alone. In addition, PR+ESWT+BA (one RCT; MD = 4.4, 95% CI: 1.6, 7.2, p = 0.51) was more effective than PR+ESWT, and PR+ESWT+BA (four RCTs; MD = 4.6, 95% CI: 1.1, 8.1, p < 0.00001) was superior to PR+BA. With regard to the MBI scores, the following interventions were observed to yield enhanced outcomes in comparison to PR: PR+rTMS (eight RCTs; MD = 6.6, 95% CI: 0.074, 13, p < 0.00001), PR+BA (eight RCTs; MD = 9.0, 95% CI: 2.9, 15, p < 0.00001), PR+M (four RCTs; MD = 18, 95% CI: 9.5, 27, p < 0.00001), PR+ESWT+BA (two RCTs; MD = 16, 95% CI: 2.9, 30, p = 0.80) all demonstrated statistically significant superiority over PR. Furthermore, no statistically significant differences were identified in the comparisons between the remaining treatment measures. For further details, please refer to Table 3.

**Table 3:** The results of the paired meta-analysis.

Comparison	MD (95% CI)	Number of studies	Number of patients	I^2^ (%)	*p*-value
FMA-UE					
B-A	**5.6 (0.90, 10)**	2	124	43.7%	0.39
C-A	2 (−8.0, 12)	1	83	-	-
D-A	−7.3 (−19, 5.1)	1	40	-	-
J-A	2.3 (−19, 24)	1	14	-	-
K-A	7.7 (−10, 25.)	1	22	-	-
L-A	**7.2 (4.4, 9.9)**	10	362	61.7%	-
M-A	**7.2 (2.4, 12)**	2	100	40.0%	-
N-A	**12 (5.6, 18)**	2	148	62.4%	-
O-A	**6.1 (4.0, 8.2)**	11	851	90.9%	0.68
P-A	**7.2 (3.9, 11)**	5	472	38.9%	-
R-A	−3.2 (−20, 14)	1	33	-	-
T-A	**7.5 (2.8, 12)**	2	100	88.6%	0.15
F-B	**11 (3.1, 19)**	1	60	-	-
G-B	9.7 (−6.1, 26)	1	40	-	-
O-B	3.4 (−1.5, 8.2)	2	120	72.8%	0.30
I-C	**8.3 (1.0, 16)**	1	100	-	-
N-C	6.0 (−3.6, 15)	1	87	-	-
P-D	**16 (5.1, 27)**	1	40	-	-
G-E	5.3 (−2.4, 13)	1	62	-	-
L-J	−2.5 (−19., 14)	1	14	-	-
O-M	−0.92 (−8, 6.1)	1	40	-	0.97
T-M	**4.4 (1.6, 7.2)**	1	40	-	0.51
U-N	5.9 (−1.3, 13)	1	50	-	-
S-O	2.5 (−4.5, 9.3)	1	40	-	-
T-O	**4.6 (1.1, 8.1)**	4	230	0.0%	-
MBI					
D-A	−2.8 (−21, 16)	1	40	-	-
K-A	−9.2 (−29, 10)	1	32	-	0.70
L-A	**6.6 (0.074, 13)**	8	329	78.4%	-
M-A	11 (−2.7, 24)	2	100	44.6%	-
N-A	0.70 (−16, 18)	1	60		-
O-A	**9.0 (2.9, 15)**	8	557	86.4%	-
P-A	**18 (9.5, 27)**	4	260	99.9%	-
T-A	**16 (2.9, 30)**	2	100	91.4%	0.80
F-B	12 (−5.9, 30)	1	69	-	-
G-B	10 (−11, 31)	1	40	-	-
H-B	8.3 (−3.7, 20)	2	130	0.0%	-
O-B	8.7 (−8.3, 26)	1	60	-	-
P-D	8.4 (−9.8, 26)	1	40	-	-
G-E	8.3 (−8.9, 26)	1	62	-	-
L-J	−2.8 (−25., 19)	1	60	-	-
Q-J	10 (−11, 32)	1	60	-	-
Q-L	13 (−8.5, 34)	1	60	-	-
O-M	−6.7 (−30, 16)	1	40	-	0.59
T-M	8.9 (−17, 34)	1	40	-	0.81
U-N	10 (−7.4, 28)	1	50	-	-
S-O	11 (−1.8, 23)	2	76	0.0%	-
T-O	9.3 (−1.5, 20)	3	170	14.5%	-

A, Physical rehabilitation; B, Body acupuncture; C, Electro-acupuncture; D, Massage; E, Proprioceptive Neuromuscular Facilitation; F, Body acupuncture plus extracorporeal shock wave treatment; G, Body acupuncture plus proprioceptive neuromuscular facilitation; H, Body acupuncture plus massage; I, Electro-acupuncture plus repetitive transcranial magnetic stimulation; J, Physical rehabilitation plus continuous theta burst stimulation; K, Physical rehabilitation plus intermittent theta burst stimulation; L, Physical rehabilitation plus repetitive transcranial magnetic stimulation; M, Physical rehabilitation plus extracorporeal shock wave treatment; N, Physical rehabilitation plus electro-acupuncture; O, Physical rehabilitation plus body acupuncture; P, Physical rehabilitation plus massage; Q, Physical rehabilitation plus repetitive transcranial magnetic stimulation plus continuous theta burst stimulation; R, Physical rehabilitation plus repetitive transcranial magnetic stimulation plus intermittent theta burst stimulation; S, Physical rehabilitation plus repetitive transcranial magnetic stimulation plus body acupuncture; T, Physical rehabilitation plus extracorporeal shock wave treatment plus body acupuncture; U, Physical rehabilitation plus repetitive transcranial magnetic stimulation plus electro-acupuncture; FMA-UE, The Fugl-Meyer Assessment-Upper Extremity scale; MBI, The Modified Barthel Index scale. Bold values indicate statistically significant results (95% CI excluding zero).

### 3.4 Network meta-analysis

The transferability hypothesis was evaluated by means of a comparison of the FMA-UE baseline data. The results demonstrated MD = −0.0609, with 95% CI [−0.2749; 0.1531], and p = 0.5772 > 0.05. This indicates that there was no statistically significant difference in the baseline FMA-UE scores among the included studies, and thus no heterogeneity. Similarly, a comparison of the MBI baseline data revealed MD = −0.1220, with 95% CI [−0.5859; 0.3419], and p = 0.6063 > 0.05. This indicates that no significant heterogeneity was detected between the MBI baseline data. In light of these findings, it can be concluded that the transferability hypothesis is supported, indicating that the baseline characteristics across different studies are comparable. This provides support for the reliability of the study outcomes.

The inconsistency tests for the FMA-UE and MBI scores yielded p -values of 0.7784 and 0.6056, respectively, both greater than 0.05. Consequently, a consistency model was selected for subsequent analysis. To further investigate the potential for internal distribution inconsistency, a node-splitting method was employed for additional testing. The forest plots demonstrate that there are no statistically significant differences between the direct and indirect comparisons at each split node ( p > 0.05), indicating that there is no evidence of inconsistency (shown in Supplementary Figure S2). In the closed-loop inconsistency test, all 95% CIs were found to include 0, indicating a high degree of consistency in the closed-loop comparisons (shown in Supplementary Table S2). Furthermore, the Brooks-Gelman-Rubin diagnostic plots indicated that the median and 97.5th percentile of the shrinkage factor exhibited a tendency toward 1 and reached a stable state after 5,000 iterations. Subsequently, the Bayesian model computations were completed with 20,000 iterations, as illustrated in Supplementary Figure S3. Furthermore, the trajectory and density plots of the model were analyzed (shown in Supplementary Figure S4). These results consistently indicate that the model exhibited excellent convergence.

**Figure 2:**
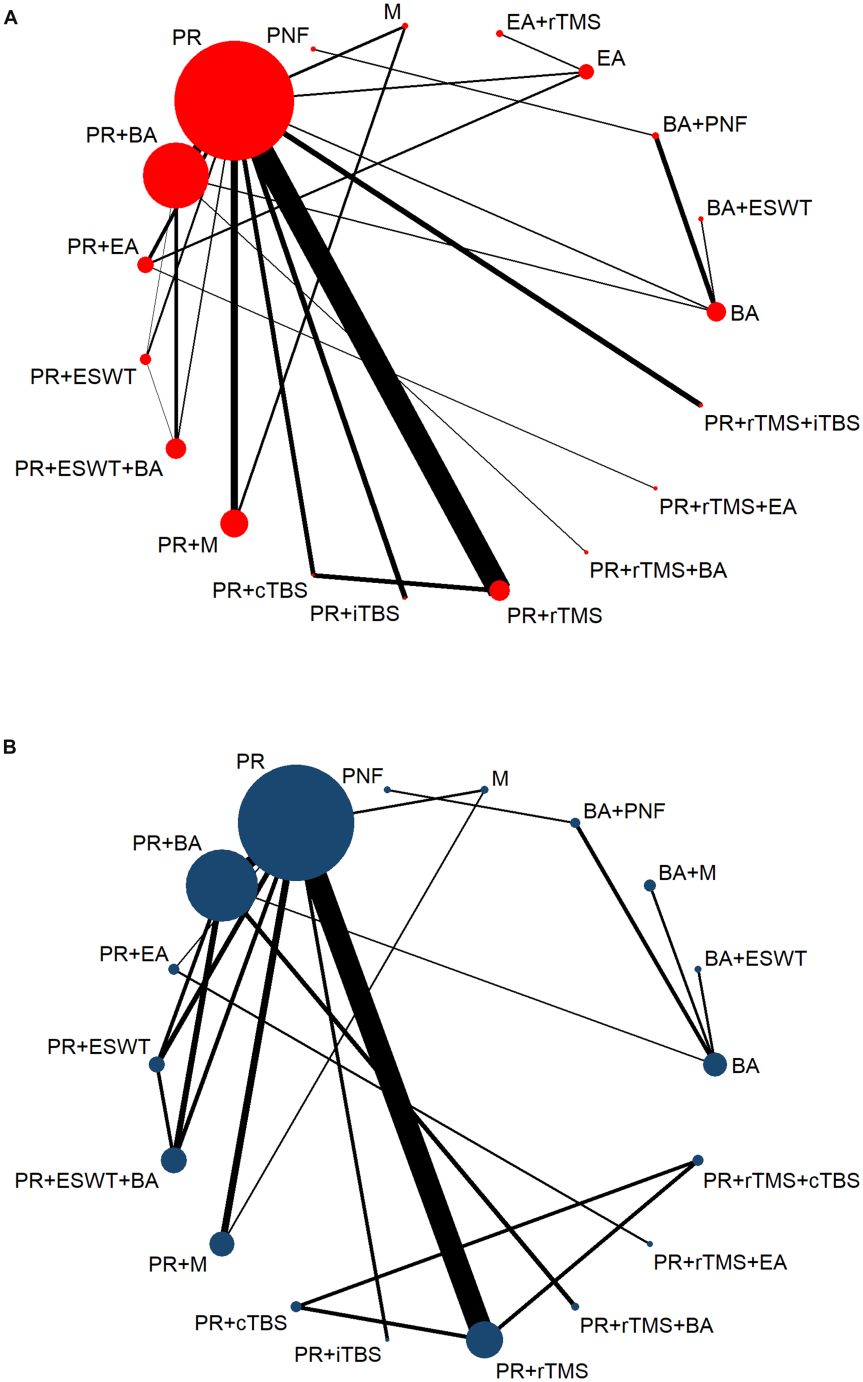
Network evidence diagram. PR, Physical rehabilitation; BA, Body acupuncture; EA, Electro-acupuncture; M, Massage; PNF, Proprioceptive Neuromuscular Facilitation; BA+ESWT, Body acupuncture plus extracorporeal shock wave treatment; BA+PNF, Body acupuncture plus proprioceptive neuromuscular facilitation; BA+M, Body acupuncture plus massage; EA+rTMS, Electro-acupuncture plus repetitive transcranial magnetic stimulation; PT+cTBS, Physical rehabilitation plus continuous theta burst stimulation; PT+iTBS, Physical rehabilitation plus intermittent theta burst stimulation; PT+rTMS, Physical rehabilitation plus repetitive transcranial magnetic stimulation; PT+ESWT, Physical rehabilitation plus extracorporeal shock wave treatment; PT+EA, Physical rehabilitation plus electro-acupuncture; PT+BA, Physical rehabilitation plus body acupuncture; PT+M, Physical rehabilitation plus massage; PT+rTMS+cTBS, Physical rehabilitation plus repetitive transcranial magnetic stimulation plus continuous theta burst stimulation; PT+rTMS+iTBS, Physical rehabilitation plus repetitive transcranial magnetic stimulation plus intermittent theta burst stimulation; PT+rTMS+BA, Physical rehabilitation plus repetitive transcranial magnetic stimulation plus body acupuncture; PT+ESWT+BA, Physical rehabilitation plus extracorporeal shock wave treatment plus body acupuncture; PT+rTMS+EA, Physical rehabilitation plus repetitive transcranial magnetic stimulation plus electro-acupuncture; FMA-UE, The Fugl-Meyer Assessment-Upper Extremity scale; MBI, The Modified Barthel Index scale. **(A)** The Fugl-Meyer Assessment-Upper Extremity scale (FMA-UE). **(B)** The Modified Barthel Index scale (MBI).

Figures 2A,B present the NMA diagrams for the impact of different treatments on FMA-UE and MBI scores, respectively. The size of the nodes in the diagrams is proportional to the number of participants in each intervention, while the thickness of the lines between nodes is proportional to the number of studies that have been conducted to make the corresponding comparisons. The largest sample sizes were observed for the PR, PR+BA, and PR+M interventions. The most frequently compared pairs were PR vs. PR+rTMS and PR vs. PR+BA.

A league table (Supplementary Table S7) provides a summary of the comparative results of different treatment methods. The table presents the treatment effects based on FMA-UE scores in the lower triangular area and the results related to MBI scores in the upper triangular area. In order to evaluate the efficacy of treatments in improving upper limb motor function, this study compared the effects of standalone PR with various combined treatment. The findings demonstrate that the combined treatments, when compared to the standalone PR, yielded significantly enhanced outcomes in terms of FMA-UE scores. The combined treatments BA+ESWT (MD = −15.15, 95% CI: −23.75, −6.48), EA+rTMS (MD = −12.11, 95% CI: −23.18, −1.07), PR+EA (MD = −11.66, 95% CI: −17.69, −5.56), and PR+rTMS+EA (MD = −17.57, 95% CI: −27.15, −7.97) were able to increase the scores by more than 10 points. Further analysis indicates that, in comparison to the BA treatment alone, the treatments of BA+ESWT (MD = −10.76, 95% CI: −18.7, −3.09) and PR+rTMS+EA (MD = −13.12, 95% CI: −23.51, −2.78) demonstrated more pronounced improvements in FMA-UE scores. Similarly, the combination of PR+ rTMS+EA (MD = −13.68, 95% CI: −24.84, −2.75) also demonstrated superior therapeutic efficacy in comparison to EA treatment alone. Furthermore, the treatments of BA+ESWT (MD = −23.16, 95% CI: −35.43, −10.96), BA+PNF (MD = −21.79, 95% CI: −40.12, −2.79), EA+rTMS (MD = −20.04, 95% CI: −33.92, −6.17), PR+rTMS (MD = −15.11, 95% CI: −24.11, −5.86), PR+rTMS (MD = −14.97, 95% CI: −24.64, −5.16), PR+EA (MD = −19.58, 95% CI: −30.17, −8.97), PR+BA (MD = −14.15, 95% CI: −22.88, −5.13), PR+M (MD = −15.21, 95% CI: −23.7, −6.52), PR+rTMS+BA (MD = −16.69, 95% CI: −27.91, −5.3), PR+ESWT+BA (MD = −17.86, 95% CI: −27.17, −8.45), and PR+rTMS+EA (MD = −25.48, 95% CI: −38.2, −12.58) all showed significant improvements in FMA-UE scores compared to M. Similarly, the combination of PR+rTMS+EA (MD = −10.41, 95% CI: −20.38, −0.45) demonstrated superior efficacy compared to PR+rTMS. The combination of PR+rTMS+EA (MD = −11.36, 95% CI: −21.23, −1.57) demonstrated a superior therapeutic effect compared to PR+BA. The combination of PR+rTMS+EA (MD = −10.3, 95% CI: −20.29, −0.1) demonstrated superior efficacy compared to PR+M, while the combination of PR+rTMS+EA (MD = −20.21, 95% CI: −39.79, −0.81) exhibited enhanced effectiveness compared to PR+rTMS+ITBS. However, when compared to BA, M was observed to have a slightly lesser impact on the total FMA score (MD = 12.33, 95% CI: 2.72 to 21.78). Similarly, PR+BA was less effective in increasing the FMA-UE score than BA+ESWT (MD = 9.01, 95% CI: 0.32 to 17.66).

In terms of enhancing patients’ capacity to perform activities of daily living, combined treatment exhibited greater efficacy than did the use of PR alone. Specifically, PR+M (MD = 18.12, 95% CI: 9.33, 26.7), PR+rTMS+BA (MD = 19.7, 95% CI: 5.77, 33.59), and PR+ESWT+BA (MD = 17.32, 95% CI: 7.32, 27.63) were all significantly more efficacious than PR alone. Further comparison revealed that the PR+M (MD = 27.19, 95% CI: 5.68, 48.22), PR+rTMS+cTBS (MD = 28.88, 95% CI: 0.29, 57.68), PR+rTMS+BA (MD = 28.75, 95% CI: 4.79, 53.01), and PR+ESWT+BA (MD = 26.42, 95% CI: 4.16, 48.75) all demonstrated superiority over the PR+iTBS. Furthermore, the PR+M (MD = 11.51, 95% CI: 0.3, 22.18) exhibited superior outcomes in comparison to the PR+rTMS.

**Figure 3:**
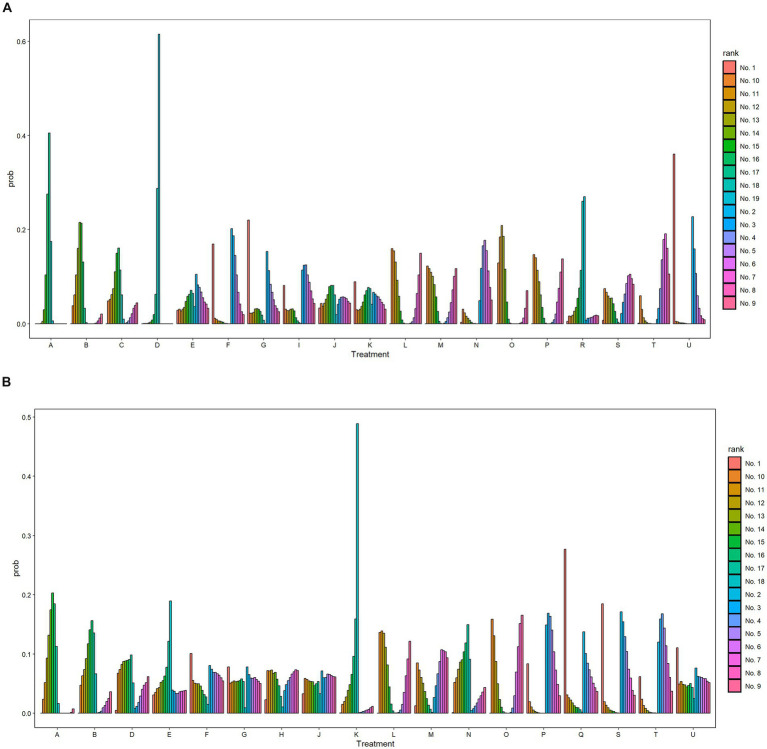
Probability ranking diagram. A, Physical rehabilitation; B, Body acupuncture; C, Electro-acupuncture; D, Massage; E, Proprioceptive Neuromuscular Facilitation; F, Body acupuncture plus extracorporeal shock wave treatment; G, Body acupuncture plus proprioceptive neuromuscular facilitation; H, Body acupuncture plus massage; I, Electro-acupuncture plus repetitive transcranial magnetic stimulation; J, Physical rehabilitation plus continuous theta burst stimulation; K, Physical rehabilitation plus intermittent theta burst stimulation; L, Physical rehabilitation plus repetitive transcranial magnetic stimulation; M, Physical rehabilitation plus extracorporeal shock wave treatment; N, Physical rehabilitation plus electro-acupuncture; O, Physical rehabilitation plus body acupuncture; P, Physical rehabilitation plus massage; Q, Physical rehabilitation plus repetitive transcranial magnetic stimulation plus continuous theta burst stimulation; R, Physical rehabilitation plus repetitive transcranial magnetic stimulation plus intermittent theta burst stimulation; S, Physical rehabilitation plus repetitive transcranial magnetic stimulation plus body acupuncture; T, Physical rehabilitation plus extracorporeal shock wave treatment plus body acupuncture; U, Physical rehabilitation plus repetitive transcranial magnetic stimulation plus electro-acupuncture; FMA-UE, The Fugl-Meyer Assessment-Upper Extremity scale; MBI, The Modified Barthel Index scale. **(A)** The Fugl-Meyer Assessment-Upper Extremity scale (FMA-UE). **(B)** The Modified Barthel Index scale (MBI).

The SUCRA values for each intervention method were calculated in order to facilitate a probabilistic ranking. The specific data can be found in Supplementary Table S3 and Supplementary Figure S5. A probability rank histogram was constructed for the purpose of visually presenting these rankings. As illustrated in Figure 3A, the three most efficacious treatment modalities for enhancing FMA-UE scores were PR+rTMS+EA (91.1%), BA+ESWT (84%), and BA+PNF (74.8%). Figure 3B illustrates that the most efficacious three treatment protocols for enhancing patients’ activities of daily living and increasing MBI scores were PR+rTMS+BA (83.1%), PR+M (80.6%), and PR+rTMS+cTBS (79.0%). Furthermore, probability rank graphs and tables were constructed, and their outcomes corroborated those of the probability rank histograms, thus providing additional validation of the analytical findings.

### 3.5 Adverse effect

A total of 16 studies (32.65%) reported adverse reactions among the 49 included studies. Twelve of the studies indicated that no adverse reactions were observed during the course of treatment. One study reported that a very small number of patients developed mild subcutaneous bruising following electroacupuncture therapy. However, these cases resolved spontaneously without the need for specialized treatment. Additionally, three studies indicated that patients experienced discomfort at the site of treatment during extracorporeal stimulation physical therapy. However, no further adverse reactions of a serious nature were reported (shown in Supplementary Table S5). Overall, extracorporeal stimulation physical therapy appears to have a favorable safety profile. Nevertheless, given the paucity of current research data, a cautious evaluation of its long-term safety and efficacy is still warranted.

### 3.6 Publication bias

**Figure 4:**
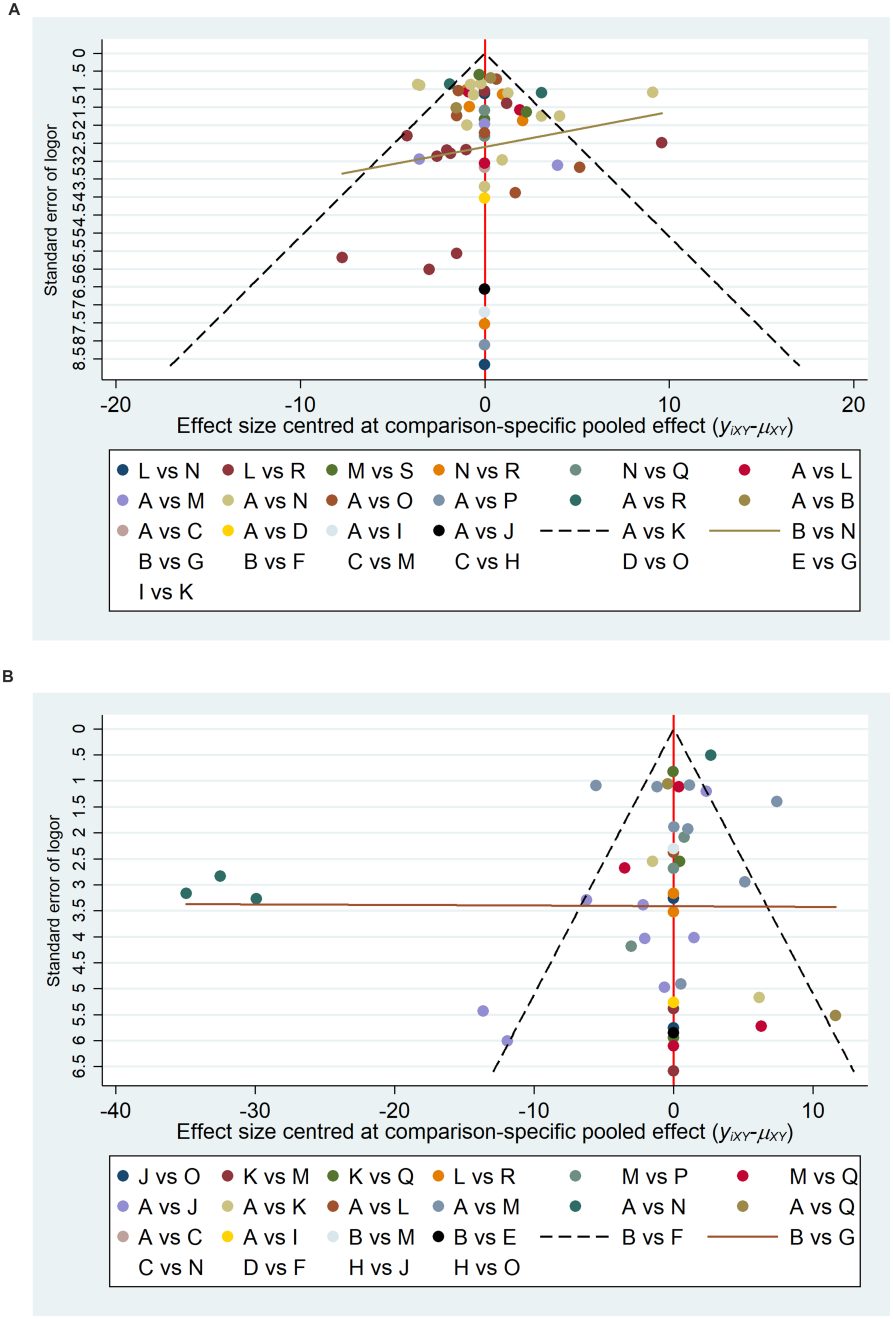
Comparative adjustment funnel plots. A, Physical rehabilitation; B, Body acupuncture; C, Electro-acupuncture; D, Massage; E, Proprioceptive Neuromuscular Facilitation; F, Body acupuncture plus extracorporeal shock wave treatment; G, Body acupuncture plus proprioceptive neuromuscular facilitation; H, Body acupuncture plus massage; I, Electro-acupuncture plus repetitive transcranial magnetic stimulation; J, Physical rehabilitation plus continuous theta burst stimulation; K, Physical rehabilitation plus intermittent theta burst stimulation; L, Physical rehabilitation plus repetitive transcranial magnetic stimulation; M, Physical rehabilitation plus extracorporeal shock wave treatment; N, Physical rehabilitation plus electro-acupuncture; O, Physical rehabilitation plus body acupuncture; P, Physical rehabilitation plus massage; Q, Physical rehabilitation plus repetitive transcranial magnetic stimulation plus continuous theta burst stimulation; R, Physical rehabilitation plus repetitive transcranial magnetic stimulation plus intermittent theta burst stimulation; S, Physical rehabilitation plus repetitive transcranial magnetic stimulation plus body acupuncture; T, Physical rehabilitation plus extracorporeal shock wave treatment plus body acupuncture; U, Physical rehabilitation plus repetitive transcranial magnetic stimulation plus electro-acupuncture; FMA-UE, The Fugl-Meyer Assessment-Upper Extremity scale; MBI, The Modified Barthel Index scale. **(A)** The Fugl-Meyer Assessment-Upper Extremity scale (FMA-UE). **(B)** The Modified Barthel Index scale (MBI).

To further investigate the potential publication bias and the impact of small sample sizes on the FMA-UE and MBI scores, corresponding funnel plots were constructed for analysis. As can be observed in Figures 4A,B, the adjusted funnel plots for the comparison of the FMA-UE and MBI scales both demonstrate a symmetrical distribution, with the majority of study points situated equidistant from the central guiding line on either side. This suggests that the included studies have moderate sample sizes and a low risk of publication bias.

### 3.7 Evidence assessment of outcome measures

Following an assessment of the pertinent outcomes using the GRADE scoring system, it was determined that the strength of evidence for the two scales under discussion ranges from very low to moderate. The principal factors responsible for the reduction in the quality of the evidence are the limitations of the study design and the considerable statistical heterogeneity. The detailed information can be found in the Supplementary Table S6.

## 4 Discussion

This study used systematic review and meta-analysis methods to thoroughly investigate the impact of different interventions on improving upper limb function in stroke patients. The results showed that a variety of combined treatment regimens significantly outperformed PR alone in improving FMA-UE scores and MBI scores.

In terms of improving upper limb motor function, the efficacy of combined treatments such as PR in conjunction with rTMS, ESWT, and EA, among others, is superior to that of standalone PR. This indicates that combined treatment strategies have significant advantages in enhancing upper limb motor function in stroke patients. Among these, the PR+rTMS+EA (MD = −17.57, 95% CI: −27.15, −7.97, SUCRA = 91.1%) regimen has demonstrated the most outstanding performance in increasing the FMA-UE score. This regimen incorporates three distinct treatment modalities: PR, rTMS, EA. These three approaches may act on the central and peripheral nervous systems through different mechanisms, resulting in additive or synergistic effects that promote the recovery of upper limb motor function. rTMS can modulate the release and expression of neurotransmitters, regulate the excitability of the cerebral cortex, and improve neural inflammation by modulating the activation and polarization of astrocytes and microglia (61). This may facilitate the reorganization of damaged neural networks and enhance motor control abilities. Electroacupuncture has been demonstrated to enhance the area of cerebral infarction and downregulate the expression of various inflammatory factors by stimulating specific acupoints, thereby further promoting neural repair and regeneration (62). Furthermore, a meta-analysis (63) has demonstrated that the combination of EA and rehabilitation training represents an efficacious approach to the reduction of post-stroke limb spasticity.

The NMA also demonstrated that the combination of PR with rTMS and BA yielded the most favorable outcomes for improving activities of daily living (MD = 19.7, 95% CI: 5.77, 33.59, SUCRA = 83.1%). Concurrently, combined treatment regimens, including PR+M (MD = 18.12, 95% CI: 9.33, 26.7, SUCRA = 80.6%) and PR+rTMS+cTBS (MD = 28.88, 95% CI: 0.29, 57.68, SUCRA = 79.0%), demonstrated remarkable efficacy, exhibiting superior outcomes compared to standalone physical therapy. This may be due to the fact that these combined treatment strategies can facilitate functional recovery through a number of different mechanisms. To illustrate, rTMS has been demonstrated to stimulate the cerebral cortex (61), thereby promoting neural remodeling. Meanwhile, BA has been shown to alleviate muscle spasticity and improve joint mobility (64). M has been demonstrated to enhance blood circulation and relieve muscle tension (65). Furthermore, the combination of rTMS+cTBS has the capacity to stimulate multiple brain regions simultaneously, thereby producing a broader neural effect (66, 67). It can therefore be concluded that these combined treatment strategies can complement each other, generating a synergistic effect and thus more effectively improving activities of daily living.

The results clearly demonstrate that combined treatments have a significant impact on patients’ motor function and also markedly enhance their activities of daily living. The combined treatment strategies have a positive impact on patients through multifaceted functional improvements, including but not limited to increasing muscle strength, improving joint mobility, enhancing coordination and balance, as well as promoting neuroplasticity and functional recovery. In contrast, a standalone physical therapy program may not address all the issues that patients face in a comprehensive manner. It can therefore be concluded that combined treatment regimens, which adopt a more comprehensive approach, represent a superior choice for enhancing patients’ activities of daily living.

It is notable that this study did not identify any significant heterogeneity during the analysis phase. This suggests that the baseline characteristics across the various studies were comparable, thereby reinforcing the reliability of the research findings. Furthermore, inconsistency tests and closed-loop inconsistency tests revealed that the model demonstrated a high degree of consistency, thereby providing additional validation for the stability of the analysis results.

Adverse events were infrequent and generally mild. Only one study reported subcutaneous bruising associated with EA, while three studies utilizing ESWT or rTMS noted transient discomfort at the stimulation site. No serious or persistent adverse events were documented, underscoring the safety of these interventions.

Follow-up outcomes demonstrated short-term rTMS efficacy (improved MBI/MAS at 2–4 weeks) and sustained motor benefits at 3 months with lower relapse rates, though ADL gains were inconsistent; PRO scores improved significantly across groups. The inconsistent ADL improvements may reflect differences in rehabilitation intensity or patient-specific functional goals.

Nevertheless, given the limited nature of the current research data, further investigation is required to evaluate the long-term safety and effectiveness of this approach.

### 4.1 Limitations and future directions

This study has several limitations that warrant consideration. First, the generalizability of findings may be constrained by geographic bias, as over 85% of included trials were conducted in China. Regional variations in rehabilitation paradigms—such as the prevalent integration of acupuncture in Chinese practice (68, 69) versus Western preferences for botulinum toxin or robotic-assisted training (70)—may introduce cultural specificity. Future multinational studies are needed to validate the cross-cultural applicability of these interventions.

Methodological shortcomings in primary studies further limit evidence quality, including inadequate allocation concealment, insufficient blinding of assessors, and incomplete reporting of prospective protocols. To address this, future studies should prioritize rigorous methodologies, including robust allocation concealment, blinded outcome assessment, and adherence to CONSORT guidelines.

Heterogeneity in intervention parameters (e.g., rTMS intensity thresholds, acupuncture session duration) complicates direct comparisons. Standardization of intervention protocols—particularly for multimodal combinations—is critical to enhance reproducibility and cross-study comparability. Additionally, the predominance of short-term interventions (≤4 weeks in 85.7% of trials) and paucity of longitudinal follow-up data (≥6 months) constrain assessment of sustained treatment effects. Prolonged observation periods are essential to evaluate durability of therapeutic benefits and monitor potential late-onset complications.

The rehabilitation process following stroke is categorized into three distinct phases—acute, subacute, and chronic—each necessitating tailored therapeutic strategies. However, this study was unable to conduct stratified analyses across these phases or specific patient subgroups due to insufficient raw data, which may partially explain the observed heterogeneity in outcomes. This limitation likely stems from a paucity of clinical trials investigating personalized, advanced rehabilitation techniques optimized for distinct recovery stages. Future research should address this gap by conducting granular analyses stratified by stroke phase, etiology (ischemic vs. hemorrhagic), and spasticity severity to identify phase-specific optimal interventions. Such stratified investigations are critical for advancing precision rehabilitation protocols in stroke care.

Mechanistically, the neuroplastic effects of combined therapies remain incompletely understood. Multicenter trials integrating advanced neuroimaging techniques (e.g., fMRI, DTI) and biomarker profiling are warranted to map neural reorganization pathways and optimize synergistic mechanisms.

Consequently, a cautious approach should be employed when interpreting the results of this NMA.

## 5 Conclusion

The findings of this study suggest that, in comparison to single treatment modalities, combined therapies are more efficacious in improving motor dysfunction and markedly enhancing the daily living abilities of patients with upper limb spasticity following a stroke. The combined physical rehabilitation and rTMS and EA appear to offer a notable advantage in terms of increasing FMA-UE scores and alleviating upper limb spasticity. The combination of physical rehabilitation with rTMS and BA may represent the optimal treatment approach for enhancing functional outcomes as measured by the MBI and improving patients’ daily living abilities. Nonetheless, it is imperative to exercise caution when interpreting these findings, given the limited number and questionable quality of the extant studies. A future imperative is to undertake additional high-quality studies that will facilitate the validation of the findings and the further exploration of the long-term efficacy and safety of combination therapies.

## Data Availability

The original contributions presented in the study are included in the article/Supplementary material, further inquiries can be directed to the corresponding author.
