# Supplementary material for: Comparison of the effects of different physical stimulation therapies on reducing upper limb spastic paralysis and motor dysfunction in stroke survivors after stroke: a network meta-analysis of randomized controlled trials
Source: Front Neurol. 2025 Apr 15;16:1554583. doi: 10.3389/fneur.2025.1554583 (PMC12037403; doi:10.3389/fneur.2025.1554583)
Supplement: Supplementary file 1 [file Data_Sheet_1.docx]

***Supplementary Material***

***Comparison of the effects of different physical stimulation therapies on reducing upper limb spastic paralysis and motor dysfunction in stroke survivors after stroke: A network meta-analysis of randomized controlled trials***

- **Table S1** Intervention Parameters and Implementation Details 3
- **Table S2** The results of the closed-loop inconsistency test 9
- **Table S3** Estimated Probability-Based Treatment Ranking Table 10
- **Table S4** Predictive Probability-Based Treatment Ranking Table 11
- **Table S5** The summary of Adverse Effects 12
- **Table S6** The results of Evidence Assessment 13
- **Table S7** Network meta-analysis results of FMA-UE and MBI 15

**Supplementary Table S1:** **Intervention Parameters and Implementation Details.**

| **Author** | **Year** | **Country** | **Intervention Group** | | | | **Control Group** | | | | **Follow-up** |
| --- | --- | --- | --- | --- | --- | --- | --- | --- | --- | --- | --- |
|  |  |  | **Intervention Details** | **Key Parameters** | **Duration per treatment** | **Frequency** | **Intervention Details** | **Key Parameters** | **Duration per treatment** | **Frequency** |  |
| **Ai YX** | 2023 | China | PR+ESWT+BA | ESWT: 8Hz, Hand: 1.0-2.0 bar; Elbow and Shoulder: 2.0-3.0 bar | ESWT: NA | ESWT: 1/4day | PR | Physical therapy, Occupational therapy | 40 min | 1/day | NA |
|  |  |  | PR+BA | BA: Upper limb acupoints, Scalp acupoints | BA: 1-15 min | BA: 1/day |  |  |  |  |  |
| **Bao YH** | 2012 | China | PR+EA | - | 75 min | 1/day | PR | Motor rehabilitation training, Bobath hand grasp | 45 min | 1/day | NA |
|  |  |  | EA | 2Hz, Upper limb acupoints | 30 min | 1/day |  |  |  |  |  |
| **Barros G** | 2014 | Brazil | PR+rTMS | rTMS: 1 Hz, 90% RMT, M1 contralesional | rTMS: NA | 3/week | PR | Motor rehabilitation training | 30 min | 3/week | After 4 weeks |
| **Chen DY** | 2024 | China | PR+ESWT | ESWT: 8Hz, Upper limb: 3.0 bar | ESWT: NA | 2/week | PR | Motor rehabilitation training, Occupational therapy | 45 min | 6/week | NA |
| **Chen QF** | 2021 | China | PR+rTMS | rTMS: 1 Hz, 90% RMT, M1 contralesional | rTMS: 20 min | 5/week | PR | Motor rehabilitation training | 30 min | 5/week | NA |
| **Chen Y** | 2021 | China | PR+iTBS | iTBS: 80% AMT, ipsilesional lateral cerebellum | iTBS: NA | 1/day | PR | Motor rehabilitation training | 50 min | 1/day | NA |
| **Chen YJ** | 2019 | Taiwan, China | PR+iTBS | iTBS: 5 Hz, 80% AMT, psilesional primary motor cortex (M1) responsible for hand movement | iTBS: NA | 5/week | PR | Physical therapy, Occupational therapy | 90 min | 5/week | NA |
| **Chu GX** | 2009 | China | PR+EA | EA: 50-100Hz, Upper limb and lower limb acupoints | EA: 20 min | EA: 6/week | PR | Motor rehabilitation training | 30 min | 3/week | NA |
| **Dang YS** | 2020 | China | PR+BA | BA: Upper limb acupoints | BA: 20 min | 1/day | PR | Motor rehabilitation training, Occupational therapy | 40 min | 1/day | NA |
| **Gu YL** | 2018 | China | PR+M | M: Upper limb and back | M: 30 min | 1/day | PR | Motor rehabilitation training | 30 min | 1/day | NA |
| **Hao JB** | 2016 | China | PR+M | M: Upper limb | M: 30 min | 1/day | PR | Bobath therapy | 30 min | 1/day | NA |
| **Jiang YY** | 2023 | China | PR+rTMS+EA | rTMS: 1 Hz, 90% RMT, M1 contralesional; EA: Upper limb acupoints | rTMS: 20 min; EA: 30 min | 5/week | PR+EA | PR: Motor rehabilitation training, Occupational therapy | PR: NA | 5/week | NA |
| **Kuzu Ö** | 2021 | Turkey | PR+rTMS | rTMS: 1 Hz, 90% RMT, M1 contralesional | rTMS: 20 min | 1/week | PR | Physical therapy, Occupational therapy | 60 min | 1/week | After 4 weeks |
|  |  |  | PR+cTBS | cTBS: 50 Hz, 80% AMT, M1 contralesional | cTBS: 40 s | 1/week |  |  |  |  |  |
| **Lei JF** | 2024 | China | PR+rTMS+BA | rTMS: 1 Hz, 90% RMT, M1 contralesional; BA: Upper limb acupoints | rTMS: 20 min; BA: 30 min | 5/week | PR+BA | PR: Physical therapy, Motor rehabilitation training | PR: NA | 5/week | NA |
| **Lei M** | 2012 | China | PR+M | M: Upper limb | M: 30 min | 5/week | PR | Physical therapy, Occupational therapy | 45 min | 5/week | NA |
| **Li B** | 2021 | China | BA+M | M: Upper limb | M: 10 min | 6/week | BA | Upper limb and lower limb acupoints; Scalp acupoints | 20 min | 6/week | NA |
| **Li BJ** | 2017 | China | PR+BA | BA: Upper limb acupoints | BA: 20 min | 5/week | PR | Modified Constraint-Induced Movement Therapy | 6 h | 5/week | NA |
| **Li D** | 2021 | China | PR+rTMS+cTBS | PR: Physical therapy, Occupational therapy | PR: 40 min | 6/week | PR+rTMS | rTMS: 1 Hz, 80% RMT, M1 contralesional | rTMS: 20 min | 6/week | NA |
|  |  |  |  |  |  |  | PR+cTBS | cTBS: 50 Hz, 80% AMT, right cerebellum | cTBS: 80 s | 6/week |  |
| **Li ZW** | 2022 | China | PR+M | M: Upper limb | M: 30 min | 6/week | PR | Motor rehabilitation training | 30 min | 6/week | NA |
| **Lin FY** | 2018 | China | PR+BA | BA: Back acupoints | BA: 20 min | 5/week | PR | Motor rehabilitation training | NA | NA | NA |
| **Liu HJ** | 2023 | China | PR+BA | BA: Upper limb and lower limb acupoints; Scalp acupoints | BA: NA | 6/week | PR | Bobath therapy | 45 min | 6/week | NA |
| **Liu QQ** | 2021 | China | BA+PNF | BA: Upper limb acupoints; Scalp acupoints | BA: 30 min | 6/week | PNF | Upper limb | NA | 6/week | NA |
| **Liu SD** | 2023 | China | EA+rTMS | rTMS: 1 Hz, M1 contralesional | rTMS: 20 min | rTMS: 5/week | EA | 2～5 Hz, Upper limb acupoints | 30 min | 6/week | After 3 months |
| **Liu SH** | 2019 | China | PR+rTMS | rTMS: 1 Hz, 120% AMT, M1 contralesional | rTMS: 20 min | 6/week | PR | Physical therapy, Occupational therapy | NA | 6/week | NA |
| **Liu Y** | 2018 | China | PR+rTMS | rTMS: 1 Hz, 90% RMT, M1 contralesional | rTMS: 24 min | 5/week | PR | Motor rehabilitation training, Occupational therapy | 40 min | 5/week | NA |
| **Ma AF** | 2022 | China | PR+BA | BA: Upper limb acupoints | BA: 30 min | 5/week | PR | Motor rehabilitation training | 30 min | 5/week | NA |
| **Ma JY** | 2020 | China | BA+M | M: Upper limb | M: 10 min | 6/week | BA | Upper limb and lower limb acupoints; Scalp acupoints | 20 min | 6/week | NA |
| **Motamed V** | 2014 | Iran | PR+rTMS | rTMS: 1 Hz, 60-80% RMT, M1 contralesional | rTMS: 20 min | 3/week | PR | Motor rehabilitation training, Occupational therapy | 60 min | 3/week | NA |
| **Ni HH** | 2012 | China | PR+BA | BA: Upper limb acupoints | BA: 20 min | 1/2day | PR | Motor rehabilitation training, Occupational therapy | NA | 6/week | NA |
| **Qin Y** | 2023 | China | PR+rTMS | rTMS: 1 Hz, 90% RMT, M1 contralesional | rTMS: NA | 5/week | PR | Motor rehabilitation training | 40 min | 5/week | NA |
| **Shi J** | 2019 | China | BA+PNF | PNF: Upper limb | PNF: NA | 6/week | BA | Upper limb and lower limb acupoints; Scalp acupoints | 30 min | 6/week | NA |
| **Sun X** | 2023 | China | PR+ESWT+BA | ESWT: 8Hz, Upper limb: 1.5-2.0 bar | ESWT: NA | ESWT: 1/4day | PR+BA | PR: Physical therapy, Occupational therapy; BA: Upper limb acupoints, Scalp acupoints | PR: 1 h; BA: 30 min | 6/week | NA |
| **Sun YZ** | 2013 | China | PR+EA | EA: Upper limb acupoints | EA: 40 min | 6/week | PR | Brunnstrom therapy, Bobath therapy | NA | 6/week | NA |
| **Tong JY** | 2022 | China | PR+BA | BA: Upper limb acupoints | BA: NA | 1/day | PR | Bobath therapy | 40 min | 1/day | NA |
| **Wang CP** | 2014 | Taiwan, China | PR+rTMS+iTBS | rTMS: 1 Hz,90% RMT, M1 contralesional; iTBS: 80% AMT, ipsilesional M1 | rTMS: 10 min; iTBS: 190 s | 5/week | PR | Occupational therapy | 60 min | 5/week | After 3 months |
| **Wang J** | 2018 | China | PR+BA | PR: Bobath therapy | PR: NA | 1/day | BA | Upper limb acupoints | 20 min | 1/day | NA |
| **Wei CB** | 2021 | China | PR+ESWT+BA | - | - | - | PR | Motor rehabilitation training | 30 min | 2/day | NA |
|  |  |  | PR+ESWT | ESWT: 5Hz, Upper limb: 2.0 bar | ESWT: NA | ESWT: 1/week |  |  |  |  |  |
|  |  |  | PR+BA | BA: Upper limb acupoints | BA: 15 min | BA: 6/week |  |  |  |  |  |
| **Wen DG** | 2020 | China | PR+M | - | 80 min | 5/week | PR | Mirror therapy | 40 min | 5/week | NA |
|  |  |  |  |  |  |  | M | Upper limb | 40 min | 5/week |  |
| **Xie WX** | 2023 | China | PR+rTMS+BA | rTMS: 1 Hz, 80% RMT, M1 contralesional; BA: Upper limb and lower limb acupoints; Scalp acupoints | rTMS: 20 min; BA: 30 min | 5/week | PR+BA | PR: Physical therapy, Occupational therapy | PR: NA | 5/week | NA |
| **Xu SF** | 2016 | China | PR+BA | BA: Upper limb and lower limb acupoints; Scalp acupoints | BA: 30 min | 5/week | PR | Motor rehabilitation training | NA | 5/week | NA |
| **Xu YL** | 2010 | China | BA | Upper limb acupoints | 30 min | 5/week | PR | Bobath therapy | 1-2 h | 5/week | NA |
| **Yang NY** | 2017 | China | PR+rTMS | rTMS: 1 Hz, 90% RMT, P5 contralesional | rTMS: NA | 5/week | PR | Physical therapy, Occupational therapy | 45 min | 5/week | After 4 weeks |
| **Yang X** | 2021 | China | PR+rTMS | rTMS: 1 Hz, 90% RMT, M1 contralesional | rTMS: 24 min | 5/week | PR | Motor rehabilitation training | 30-40 min | 5/week | NA |
| **Zhang L** | 2015 | China | PR+BA | BA: Upper limb acupoints | BA: 20 min | 1/day | PR | Bobath therapy | NA | NA | NA |
| **Zhang QF** | 2021 | China | PR+M | M: Upper limb | M: 15 min | 1/day | PR | Motor rehabilitation training | 40 min | 1/day | NA |
| **Zhang X** | 2021 | China | PR+ESWT+BA | ESWT: 8Hz, Upper limb: 2.0-2.5 bar | ESWT: NA | ESWT: 1/week | PR+BA | PR: Physical therapy, Occupational therapy; BA: Upper limb acupoints | PR: 120 min; BA: 30 min | 5/week | After 3 months |
| **Zhao J** | 2021 | China | PR+rTMS | rTMS: 1 Hz, 80% RMT, M1 contralesional | rTMS: NA | 6/week | PR | Motor rehabilitation training | ＞3 h | 6/week | After 2 weeks |
| **Zhao JY** | 2021 | China | BA+ ESWT | ESWT: 8Hz, Upper limb: 1.5-2.0 bar | ESWT: 25 min | ESWT: 2/week | BA | Upper limb acupoints | 30 min | 6/week | NA |
| **Zhou P** | 2019 | China | PR+BA | PR: Motor rehabilitation training, Bobath therapy | PR: 45 min | 5/week | BA | Upper limb and lower limb acupoints | NA | 5/week | NA |

PR: Physical rehabilitation; BA: Body acupuncture; EA: Electro-acupuncture; M: Massage; PNF: Proprioceptive Neuromuscular Facilitation; ESWT: Extracorporeal shock wave treatment; rTMS: repetitive Transcranial Magnetic Stimulation; cTBS: continuous Theta Burst Stimulation; iTBS: intermittent Theta Burst Stimulation.

**Supplementary Table S2: The results of the closed-loop inconsistency test.**

| **Loop** | **IF** | **seIF** | **Z_value** | **P_value** | **95%CI** | **Loop_Heterog_tau2** |
| --- | --- | --- | --- | --- | --- | --- |
| **FMA-UE** | | | | | | |
| **A-C-M** | 7.490 | 5.401 | 1.387 | 0.166 | **(0.00,18.08)** | 0.000 |
| **A-I-K** | 7.464 | 10.931 | 0.683 | 0.495 | **(0.00,28.89)** | 11.165 |
| **A-L-S** | 5.87 | 1.628 | 3.605 | 0.000 | **(2.68,9.06)** | 0.000 |
| **A-N-S** | 3.827 | 3.346 | 1.144 | 0.253 | **(0.00,10.39)** | 11.574 |
| **A-B-N** | 3.255 | 3.799 | 0.857 | 0.392 | **(0.00,10.70)** | 11.701 |
| **A-D-O** | 1.772 | 6.476 | 0.274 | 0.784 | **(0.00,14.46)** | 1.633 |
| **L-N-S** | 1.412 | 1.412 | 0.820 | 0.820 | **(0.00,4.79)** | 0.000 |
| **A-L-N** | 0.132 | 5.032 | 0.026 | 0.979 | **(0.00,10.00)** | 14.349 |
| **MBI** | | | | | | |
| **A-D-P** | 35.799 | 4.526 | 7.909 | 0.000 | **(26.93,44.67)** | 0.000 |
| **M-O-T** | 6.492 | 8.089 | 0.802 | 0.422 | **(0.00,22.35)** | 0.000 |
| **A-M-T** | 5.872 | 6.461 | 0.909 | 0.363 | **(0.00,18.53)** | 0.000 |
| **A-M-O** | 5.366 | 8.099 | 0.663 | 0.508 | **(0.00,21.24)** | 17.862 |
| **A-O-T** | 4.565 | 4.273 | 1.068 | 0.285 | **(0.00,12.94)** | 9.974 |
| **J-L-Q** | - | - | - | - | **-** | 0.000 |

*** Note: Loop J-L-Q is formed only by multi-arm trial(s) - Consistent by definition

A: Physical rehabilitation; B: Body acupuncture; C: Electro-acupuncture; D: Massage; E: Proprioceptive Neuromuscular Facilitation; F: Body acupuncture plus extracorporeal shock wave treatment; G: Body acupuncture plus proprioceptive neuromuscular facilitation; H: Body acupuncture plus massage; I: Electro-acupuncture plus repetitive transcranial magnetic stimulation; J: Physical rehabilitation plus continuous theta burst stimulation; K: Physical rehabilitation plus intermittent theta burst stimulation; L: Physical rehabilitation plus repetitive transcranial magnetic stimulation; M: Physical rehabilitation plus extracorporeal shock wave treatment; N: Physical rehabilitation plus electro-acupuncture; O: Physical rehabilitation plus body acupuncture; P: Physical rehabilitation plus massage; Q: Physical rehabilitation plus repetitive transcranial magnetic stimulation plus continuous theta burst stimulation; R: Physical rehabilitation plus repetitive transcranial magnetic stimulation plus intermittent theta burst stimulation; S: Physical rehabilitation plus repetitive transcranial magnetic stimulation plus body acupuncture; T: Physical rehabilitation plus extracorporeal shock wave treatment plus body acupuncture; U: Physical rehabilitation plus repetitive transcranial magnetic stimulation plus electro-acupuncture; FMA-UE: The Fugl-Meyer Assessment-Upper Extremity scale; MBI: The Modified Barthel Index scale.

**Supplementary Table S3: Estimated Probability-Based Treatment Ranking Table.**

| **Treatment** | **FMA-UE** | | | **MBI** | | |
| --- | --- | --- | --- | --- | --- | --- |
|  | **SUCRA** | **Pr Best** | **MeanRank** | **SUCRA** | **Pr Best** | **MeanRank** |
| **A** | 13.2 | 0.0 | 16.6 | 20.8 | 0.0 | 14.5 |
| **B** | 29.3 | 0.0 | 13.7 | 23.8 | 0.0 | 14.0 |
| **C** | 30.3 | 0.0 | 13.5 | - | - | - |
| **D** | 2.9 | 0.0 | 18.5 | 34.8 | 0.3 | 12.1 |
| **E** | 52.7 | 2.2 | 9.5 | 34.3 | 2.2 | 12.2 |
| **F** | 84.6 | 16.8 | 3.8 | 61.3 | 10.4 | 7.6 |
| **G** | 74.8 | 22.2 | 5.5 | 55.7 | 7.7 | 8.5 |
| **H** | - | - | - | 51.1 | 2.3 | 9.3 |
| **I** | 72.5 | 8.1 | 6.0 | - | - | - |
| **J** | 47.9 | 3.5 | 10.4 | 51.6 | 3.0 | 9.2 |
| **K** | 50.6 | 8.5 | 9.9 | 9.9 | 0.0 | 16.3 |
| **L** | 48.2 | 0.0 | 10.3 | 42.9 | 0.0 | 10.7 |
| **M** | 47.4 | 0.0 | 10.5 | 57.2 | 1.0 | 8.3 |
| **N** | 73.1 | 0.2 | 5.8 | 27.1 | 0.0 | 13.4 |
| **O** | 40.2 | 0.0 | 11.8 | 51.3 | 0.0 | 9.3 |
| **P** | 49.1 | 0.0 | 10.2 | 80.6 | 8.8 | 4.3 |
| **Q** | - | - | - | 79.0 | 27.0 | 4.6 |
| **R** | 17.5 | 0.6 | 15.8 | - | - | - |
| **S** | 57.3 | 0.5 | 8.7 | **83.1** | 20.1 | 3.9 |
| **T** | 67.3 | 0.1 | 6.9 | 78.1 | 6.4 | 4.7 |
| **U** | **91.1** | 37.3 | 2.6 | 57.4 | 10.7 | 8.2 |

A: Physical rehabilitation; B: Body acupuncture; C: Electro-acupuncture; D: Massage; E: Proprioceptive Neuromuscular Facilitation; F: Body acupuncture plus extracorporeal shock wave treatment; G: Body acupuncture plus proprioceptive neuromuscular facilitation; H: Body acupuncture plus massage; I: Electro-acupuncture plus repetitive transcranial magnetic stimulation; J: Physical rehabilitation plus continuous theta burst stimulation; K: Physical rehabilitation plus intermittent theta burst stimulation; L: Physical rehabilitation plus repetitive transcranial magnetic stimulation; M: Physical rehabilitation plus extracorporeal shock wave treatment; N: Physical rehabilitation plus electro-acupuncture; O: Physical rehabilitation plus body acupuncture; P: Physical rehabilitation plus massage; Q: Physical rehabilitation plus repetitive transcranial magnetic stimulation plus continuous theta burst stimulation; R: Physical rehabilitation plus repetitive transcranial magnetic stimulation plus intermittent theta burst stimulation; S: Physical rehabilitation plus repetitive transcranial magnetic stimulation plus body acupuncture; T: Physical rehabilitation plus extracorporeal shock wave treatment plus body acupuncture; U: Physical rehabilitation plus repetitive transcranial magnetic stimulation plus electro-acupuncture; FMA-UE: The Fugl-Meyer Assessment-Upper Extremity scale; MBI: The Modified Barthel Index scale.

**Supplementary Table S4: Predictive Probability-Based Treatment Ranking Table.**

| **Treatment** | **FMA-UE** | | | **MBI** | | |
| --- | --- | --- | --- | --- | --- | --- |
|  | **SUCRA** | **Pr Best** | **MeanRank** | **SUCRA** | **Pr Best** | **MeanRank** |
| **A** | 14.8 | 0.0 | 16.3 | 25.6 | 0.0 | 13.7 |
| **B** | 32.8 | 0.0 | 13.1 | 27.6 | 0.0 | 13.3 |
| **C** | 32.0 | 0.0 | 13.2 | - | - | - |
| **D** | 3.5 | 0.0 | 18.4 | 36.3 | 0.6 | 11.8 |
| **E** | 53.1 | 3.6 | 9.4 | 35.6 | 2.6 | 12.0 |
| **F** | 83.1 | 16.8 | 4.0 | 59.8 | 9.5 | 7.8 |
| **G** | 74.0 | 21.0 | 5.7 | 54.8 | 7.7 | 8.7 |
| **H** | - | - | - | 50.5 | 3.0 | 9.4 |
| **I** | 70.9 | 8.8 | 6.2 | - | - | - |
| **J** | 47.3 | 3.2 | 10.5 | 51.8 | 3.6 | 9.2 |
| **K** | 50.6 | 8.5 | 9.9 | 13.0 | 0.1 | 15.8 |
| **L** | 48.2 | 0.0 | 10.3 | 44.5 | 0.2 | 10.4 |
| **M** | 47.7 | 0.1 | 10.4 | 56.5 | 2.2 | 8.4 |
| **N** | 71.3 | 1.3 | 6.2 | 30.0 | 0.2 | 12.9 |
| **O** | 42.4 | 0.0 | 11.4 | 51.2 | 0.3 | 9.3 |
| **P** | 49.1 | 0.1 | 10.2 | 76.6 | 10.0 | 5.0 |
| **Q** | - | - | - | 76.4 | 23.3 | 5.0 |
| **R** | 18.1 | 0.5 | 15.7 | - | - | - |
| **S** | 56.8 | 0.9 | 8.8 | **78.8** | 18.2 | 4.6 |
| **T** | 64.7 | 0.4 | 7.4 | 74.3 | 8.9 | 5.4 |
| **U** | **89.4** | 34.7 | 2.9 | 56.8 | 9.7 | 8.4 |

A: Physical rehabilitation; B: Body acupuncture; C: Electro-acupuncture; D: Massage; E: Proprioceptive Neuromuscular Facilitation; F: Body acupuncture plus extracorporeal shock wave treatment; G: Body acupuncture plus proprioceptive neuromuscular facilitation; H: Body acupuncture plus massage; I: Electro-acupuncture plus repetitive transcranial magnetic stimulation; J: Physical rehabilitation plus continuous theta burst stimulation; K: Physical rehabilitation plus intermittent theta burst stimulation; L: Physical rehabilitation plus repetitive transcranial magnetic stimulation; M: Physical rehabilitation plus extracorporeal shock wave treatment; N: Physical rehabilitation plus electro-acupuncture; O: Physical rehabilitation plus body acupuncture; P: Physical rehabilitation plus massage; Q: Physical rehabilitation plus repetitive transcranial magnetic stimulation plus continuous theta burst stimulation; R: Physical rehabilitation plus repetitive transcranial magnetic stimulation plus intermittent theta burst stimulation; S: Physical rehabilitation plus repetitive transcranial magnetic stimulation plus body acupuncture; T: Physical rehabilitation plus extracorporeal shock wave treatment plus body acupuncture; U: Physical rehabilitation plus repetitive transcranial magnetic stimulation plus electro-acupuncture; FMA-UE: The Fugl-Meyer Assessment-Upper Extremity scale; MBI: The Modified Barthel Index scale.

**Supplementary Table S5: The summary of Adverse Effects.**

| **Study** | **Adverse Effects** |
| --- | --- |
| **Bao YH 2012** | In the electro-acupuncture group, subcutaneous bruising was occasionally observed following treatment. This was reported to be absorbed and resolved within two weeks, with no other adverse reactions documented. |
| **Chen Y 2021** | No adverse effects. |
| **Kuzu Ö 2021** | No adverse effects. |
| **Jiang YY 2023** | One patient in the observation group reported mild discomfort at the rTMS stimulation site during the course of treatment, which subsequently abated. |
| **Liu HJ 2023** | No adverse effects. |
| **Liu SD 2023** | No adverse effects. |
| **Ma AF 2022** | No adverse effects. |
| **Motamed V 2014** | No adverse effects. |
| **Sun X 2023** | The primary adverse reactions observed in patients were musculoskeletal discomfort at the treatment site, which was generally well-tolerated. No evidence of petechiae or ecchymosis was discernible, and no other discomfort was reported. |
| **Wang CP 2014** | No adverse effects. |
| **Wei CB 2021** | No adverse effects. |
| **Xie WX 2023** | Only a small number of patients reported discomfort at the stimulation site during the initial rTMS treatment, and no other adverse reactions were observed. |
| **Xu SF 2016** | No adverse effects. |
| **Yang NY 2017** | No adverse effects. |
| **Yang X 2021** | No adverse effects. |
| **Zhang X 2021** | No adverse effects. |

**Supplementary Table S6: The results of Evidence Assessment.**

| **Comparison** | **Risk of bias** | **Inconsistency** | **Indirectness** | **Imprecision** | **Publication Bias** | **GRADE** |
| --- | --- | --- | --- | --- | --- | --- |
| **FMA-UE** | | | | | | |
| **B-A** | Serious | Not serious | Not serious | Not serious | Not serious | MODERATE |
| **C-A** | Serious | Not serious | Not serious | Very serious | Not serious | VERY LOW |
| **D-A** | Serious | Not serious | Not serious | Very serious | Not serious | VERY LOW |
| **J-A** | Serious | Not serious | Not serious | Very serious | Not serious | VERY LOW |
| **K-A** | Serious | Not serious | Not serious | Very serious | Not serious | VERY LOW |
| **L-A** | Serious | Serious | Not serious | Not serious | Not serious | LOW |
| **M-A** | Serious | Not serious | Not serious | Not serious | Not serious | MODERATE |
| **N-A** | Serious | Serious | Not serious | Not serious | Not serious | LOW |
| **O-A** | Serious | Serious | Not serious | Not serious | Not serious | LOW |
| **P-A** | Serious | Not serious | Not serious | Not serious | Not serious | MODERATE |
| **R-A** | Serious | Not serious | Not serious | Very serious | Not serious | VERY LOW |
| **T-A** | Serious | Serious | Not serious | Not serious | Not serious | LOW |
| **F-B** | Serious | Not serious | Not serious | Serious | Not serious | LOW |
| **G-B** | Serious | Not serious | Not serious | Very serious | Not serious | VERY LOW |
| **O-B** | Serious | Serious | Not serious | Very serious | Not serious | VERY LOW |
| **I-C** | Serious | Not serious | Not serious | Not serious | Not serious | MODERATE |
| **N-C** | Serious | Not serious | Not serious | Very serious | Not serious | VERY LOW |
| **P-D** | Serious | Not serious | Not serious | Serious | Not serious | LOW |
| **G-E** | Serious | Not serious | Not serious | Very serious | Not serious | VERY LOW |
| **L-J** | Serious | Not serious | Not serious | Very serious | Not serious | VERY LOW |
| **O-M** | Serious | Not serious | Not serious | Very serious | Not serious | VERY LOW |
| **T-M** | Serious | Not serious | Not serious | Serious | Not serious | LOW |
| **U-N** | Serious | Not serious | Not serious | Very serious | Not serious | VERY LOW |
| **S-O** | Serious | Not serious | Not serious | Very serious | Not serious | VERY LOW |
| **T-O** | Serious | Not serious | Not serious | Serious | Not serious | LOW |
| **MBI** | | | | | | |
| **D-A** | Serious | Not serious | Not serious | Very serious | Not serious | VERY LOW |
| **K-A** | Serious | Not serious | Not serious | Very serious | Not serious | VERY LOW |
| **L-A** | Serious | Serious | Not serious | Not serious | Not serious | LOW |
| **M-A** | Serious | Not serious | Not serious | Very serious | Not serious | VERY LOW |
| **N-A** | Serious | Not serious | Not serious | Very serious | Not serious | VERY LOW |
| **O-A** | Serious | Serious | Not serious | Not serious | Not serious | LOW |
| **P-A** | Serious | Serious | Not serious | Not serious | Not serious | LOW |
| **T-A** | Serious | Serious | Not serious | Not serious | Not serious | LOW |
| **F-B** | Serious | Not serious | Not serious | Very serious | Not serious | VERY LOW |
| **G-B** | Serious | Not serious | Not serious | Very serious | Not serious | VERY LOW |
| **H-B** | Serious | Not serious | Not serious | Very serious | Not serious | VERY LOW |
| **O-B** | Serious | Not serious | Not serious | Very serious | Not serious | VERY LOW |
| **P-D** | Serious | Not serious | Not serious | Very serious | Not serious | VERY LOW |
| **G-E** | Serious | Not serious | Not serious | Very serious | Not serious | VERY LOW |
| **L-J** | Serious | Not serious | Not serious | Very serious | Not serious | VERY LOW |
| **Q-J** | Serious | Not serious | Not serious | Very serious | Not serious | VERY LOW |
| **Q-L** | Serious | Not serious | Not serious | Very serious | Not serious | VERY LOW |
| **O-M** | Serious | Not serious | Not serious | Very serious | Not serious | VERY LOW |
| **T-M** | Serious | Not serious | Not serious | Very serious | Not serious | VERY LOW |
| **U-N** | Serious | Not serious | Not serious | Very serious | Not serious | VERY LOW |
| **S-O** | Serious | Not serious | Not serious | Very serious | Not serious | VERY LOW |
| **T-O** | Serious | Not serious | Not serious | Very serious | Not serious | VERY LOW |

A: Physical rehabilitation; B: Body acupuncture; C: Electro-acupuncture; D: Massage; E: Proprioceptive Neuromuscular Facilitation; F: Body acupuncture plus extracorporeal shock wave treatment; G: Body acupuncture plus proprioceptive neuromuscular facilitation; H: Body acupuncture plus massage; I: Electro-acupuncture plus repetitive transcranial magnetic stimulation; J: Physical rehabilitation plus continuous theta burst stimulation; K: Physical rehabilitation plus intermittent theta burst stimulation; L: Physical rehabilitation plus repetitive transcranial magnetic stimulation; M: Physical rehabilitation plus extracorporeal shock wave treatment; N: Physical rehabilitation plus electro-acupuncture; O: Physical rehabilitation plus body acupuncture; P: Physical rehabilitation plus massage; Q: Physical rehabilitation plus repetitive transcranial magnetic stimulation plus continuous theta burst stimulation; R: Physical rehabilitation plus repetitive transcranial magnetic stimulation plus intermittent theta burst stimulation; S: Physical rehabilitation plus repetitive transcranial magnetic stimulation plus body acupuncture; T: Physical rehabilitation plus extracorporeal shock wave treatment plus body acupuncture; U: Physical rehabilitation plus repetitive transcranial magnetic stimulation plus electro-acupuncture; FMA-UE: The Fugl-Meyer Assessment-Upper Extremity scale; MBI: The Modified Barthel Index scale.

**Supplementary Table S6: Network meta-analysis results of FMA-UE and MBI.**

| **Treatment** | **MBI** | | | | | | | | | | | | | | | | | | | | |
| --- | --- | --- | --- | --- | --- | --- | --- | --- | --- | --- | --- | --- | --- | --- | --- | --- | --- | --- | --- | --- | --- |
| **FMA** | **A** | 0.29 (-18.07, 18.42) | - | 3.6 (-12.86, 19.49) | 1.96 (-31.4, 34.74) | 12.28 (-13.06, 37.85) | 10.07 (-18.09, 38.01) | 8.66 (-13.33, 30.1) | - | 9.59 (-12, 30.97) | -9.12 (-28.45, 10.43) | **6.69 (0.07, 13.19)** | 10.92 (-1.08, 23.06) | 0.84 (-16.62, 18.13) | **8.95 (2.96, 14.98)** | **18.12 (9.33, 26.7)** | 19.85 (-1.36, 40.91) | - | **19.7 (5.77, 33.59)** | **17.32 (7.32, 27.63)** | 11.12 (-13.93, 36.06) |
|  | **-4.4 (-8.16, -0.55)** | **B** | - | 3.25 (-21.11, 27.69) | 1.65 (-25.15, 28.91) | 12.14 (-5.78, 30.08) | 9.84 (-11.16, 30.75) | 8.27 (-3.8, 20.55) | - | 9.28 (-18.84, 37.65) | -9.28 (-36.48, 17.18) | 6.41 (-12.84, 25.71) | 10.74 (-10.72, 32.18) | 0.53 (-24.66, 25.73) | 8.75 (-8.77, 25.97) | 17.89 (-2.32, 37.97) | 19.75 (-8.48, 47.66) | - | 19.44 (-1.74, 40.89) | 17.17 (-2.8, 36.95) | 10.99 (-19.7, 42.21) |
|  | -3.82 (-12.05, 4.54) | 0.55 (-8.59, 9.79) | **C** | - | - | - | - | - | - | - | - | - | - | - | - | - | - | - | - | - | - |
|  | 7.96 (-0.74, 16.54) | **12.33 (2.72, 21.78)** | 11.76 (-0.16, 23.7) | **D** | -1.66 (-38.39, 35.12) | 8.79 (-21.17, 39.39) | 6.6 (-25.9, 39.06) | 5.08 (-22.05, 31.89) | - | 6.11 (-21.07, 33) | -12.54 (-37.41, 13.26) | 3.11 (-13.94, 20.78) | 7.46 (-12.4, 27.66) | -2.8 (-26.49, 21.28) | 5.44 (-11.43, 22.68) | 14.57 (-1.49, 31.06) | 16.39 (-9.88, 42.72) | - | 16.12 (-5.05, 37.6) | 13.79 (-4.86, 33.18) | 7.76 (-21.93, 37.83) |
|  | -8.69 (-26.72, 9.68) | -4.32 (-21.94, 13.59) | -4.81 (-24.5, 15.55) | -16.66 (-36.61, 3.84) | **E** | 10.41 (-21.8, 43.2) | 8.06 (-9.38, 25.69) | 6.59 (-23.32, 36.34) | - | 7.62 (-31.53, 47.1) | -10.98 (-49.06, 27.22) | 4.69 (-28.86, 38.56) | 9.11 (-25.82, 43.99) | -1.15 (-38.79, 36.49) | 7.09 (-25.37, 39.77) | 16.33 (-17.62, 50.75) | 18.06 (-21.27, 57) | - | 17.84 (-16.59, 52.97) | 15.52 (-18.33, 49.42) | 9.33 (-32.46, 51.01) |
|  | **-15.15 (-23.75, -6.48)** | **-10.76 (-18.7, -3.09)** | -11.32 (-23.58, 0.47) | **-23.16 (-35.43, -10.96)** | -6.57 (-26.05, 12.92) | **F** | -2.3 (-29.88, 24.87) | -3.84 (-25.22, 17.93) | - | -2.7 (-36.02, 30.26) | -21.41 (-53.78, 10.22) | -5.62 (-31.99, 20.6) | -1.41 (-29.29, 26.37) | -11.52 (-42.04, 18.82) | -3.26 (-28.36, 21.28) | 5.91 (-21.56, 32.44) | 7.56 (-25.92, 40.46) | - | 7.37 (-20.06, 35.16) | 5.07 (-21.69, 31.8) | -1.04 (-36.57, 34.39) |
|  | -13.96 (-30.17, 2.98) | -9.56 (-25.42, 6.8) | -10.13 (-28.23, 8.8) | **-21.79 (-40.12, -2.79)** | -5.25 (-12.82, 2.55) | 1.24 (-16.38, 19.26) | **G** | -1.55 (-25.89, 22.73) | - | -0.4 (-36.08, 35.42) | -19.08 (-53.33, 15.23) | -3.55 (-32.49, 25.53) | 0.89 (-29.22, 31.02) | -9.38 (-42.12, 23.67) | -1.23 (-28.54, 26.52) | 8.1 (-21.4, 37.94) | 9.95 (-25.59, 45.39) | - | 9.45 (-20.14, 39.78) | 7.12 (-21.82, 36.68) | 1.07 (-35.87, 39.14) |
|  | - | - | - | - | - | - | - | **H** | - | - | - | - | - | - | - | - | - | - | - | - | - |
|  | **-12.11 (-23.18, -1.07)** | -7.7 (-19.53, 4.06) | **-8.28 (-15.69, -1.03)** | **-20.04 (-33.92, -6.17)** | -3.5 (-25.12, 17.66) | 3.06 (-11.04, 17.22) | 1.76 (-18.36, 21.5) | - | **I** | 1.16 (-29.65, 32.3) | -17.61 (-46.89, 11.54) | -1.95 (-24.36, 20.82) | 2.37 (-21.82, 27.1) | -7.83 (-35.56, 20.25) | 0.34 (-20.35, 21.55) | 9.64 (-14.07, 33.17) | 11.47 (-19.41, 41.41) | - | 11.12 (-13.27, 36.02) | 8.8 (-14.03, 32.2) | 2.69 (-30.25, 36.2) |
|  | -6.71 (-20.27, 6.85) | -2.27 (-16.46, 11.75) | -2.94 (-18.82, 13.13) | -14.66 (-30.73, 1.52) | 1.89 (-21.13, 24.35) | 8.54 (-7.67, 24.71) | 7.14 (-14.35, 28.39) | - | 5.32 (-12.17, 23.01) | **J** | -18.67 (-47.38, 10.27) | -2.86 (-23.48, 17.51) | 1.27 (-23.41, 26.24) | -8.77 (-36.71, 19.2) | -0.67 (-23.08, 21.67) | 8.66 (-15.04, 31.63) | 10.25 (-9.78, 30.43) | - | 10.05 (-15.94, 35.87) | 7.73 (-16, 31.74) | 1.62 (-31.49, 34.85) |
|  | -7.84 (-25.48, 10.4) | -3.42 (-21.45, 15.2) | -4 (-23.56, 16.1) | -15.72 (-35.47, 4.41) | 0.87 (-24.52, 26.55) | 7.37 (-12.34, 27.77) | 6.15 (-18.23, 30.48) | - | 4.41 (-16.69, 25.51) | -1.13 (-23.31, 21.69) | **K** | 15.77 (-5.09, 36.08) | 19.94 (-2.94, 42.86) | 9.89 (-16.14, 35.87) | 17.96 (-2.43, 38.45) | **27.19 (5.68, 48.22)** | **28.88 (0.29, 57.68)** | - | **28.75 (4.79, 53.01)** | **26.42 (4.16, 48.75)** | 20.3 (-11.49, 51.94) |
|  | **-7.15 (-9.88, -4.33)** | -2.73 (-7.43, 1.95) | -3.3 (-12.22, 5.42) | **-15.11 (-24.11, -5.86)** | 1.59 (-16.98, 19.73) | 8.03 (-1, 17.14) | 6.78 (-10.13, 23.4) | - | 4.99 (-6.52, 16.46) | -0.39 (-13.85, 13.07) | 0.73 (-17.65, 18.71) | **L** | 4.28 (-9.19, 18) | -5.84 (-24.25, 12.73) | 2.31 (-6.64, 11.17) | **11.51 (0.3, 22.18)** | 13.25 (-6.94, 33.31) | - | 13.06 (-2.29, 28.41) | 10.69 (-1.23, 22.94) | 4.56 (-21.39, 30.52) |
|  | **-7.01 (-11.49, -2.61)** | -2.64 (-8.39, 3.04) | -3.22 (-12.72, 6.12) | **-14.97 (-24.64, -5.16)** | 1.69 (-17.13, 19.94) | 8.16 (-1.52, 17.72) | 6.91 (-10.23, 23.52) | - | 5.12 (-6.93, 17.12) | -0.36 (-14.57, 14.01) | 0.71 (-18.11, 18.95) | 0.13 (-5.21, 5.3) | **M** | -10.11 (-31.42, 10.97) | -1.97 (-14.74, 10.76) | 7.32 (-7.76, 21.97) | 9.01 (-15.46, 33.11) | - | 8.79 (-8.91, 26.3) | 6.43 (-8.08, 20.96) | 0.33 (-27.68, 27.96) |
|  | **-11.66 (-17.69, -5.56)** | **-7.27 (-14.49, -0.15)** | -7.83 (-16.18, 0.25) | **-19.58 (-30.17, -8.97)** | -3.01 (-22.38, 15.97) | 3.48 (-7.06, 14.21) | 2.28 (-15.66, 19.53) | - | 0.49 (-10.65, 11.47) | -4.93 (-19.87, 9.88) | -3.82 (-22.99, 14.94) | -4.53 (-11.15, 2.14) | -4.64 (-12.2, 2.84) | **N** | 8.1 (-10.07, 26.31) | 17.39 (-2.24, 36.34) | 19 (-8.38, 46.61) | - | 18.93 (-3.32, 40.93) | 16.56 (-3.22, 36.67) | 10.5 (-7.65, 28.55) |
|  | **-6.18 (-8.22, -4.19)** | -1.79 (-5.59, 1.98) | -2.35 (-10.97, 6.05) | **-14.15 (-22.88, -5.13)** | 2.52 (-15.85, 20.51) | **9.01 (0.32, 17.66)** | 7.76 (-9, 23.97) | - | 5.93 (-5.28, 17.2) | 0.52 (-13.11, 14.27) | 1.65 (-16.72, 19.35) | 0.96 (-2.53, 4.35) | 0.85 (-3.67, 5.45) | 5.47 (-0.89, 11.83) | **O** | 9.21 (-1.45, 19.47) | 10.88 (-10.88, 32.93) | - | 10.76 (-1.7, 23.23) | 8.42 (-1.17, 18.2) | 2.17 (-23.57, 27.93) |
|  | **-7.26 (-10.68, -3.99)** | -2.87 (-7.97, 2.16) | -3.43 (-12.47, 5.37) | **-15.21 (-23.7, -6.52)** | 1.4 (-17.33, 19.59) | 7.89 (-1.43, 17.19) | 6.64 (-10.46, 23.24) | - | 4.86 (-6.78, 16.23) | -0.53 (-14.52, 13.61) | 0.57 (-18.05, 18.59) | -0.1 (-4.65, 4.1) | -0.23 (-5.81, 5.37) | 4.43 (-2.57, 11.28) | -1.08 (-5.01, 2.71) | **P** | 1.67 (-21.05, 24.48) | - | 1.55 (-14.58, 18.12) | -0.85 (-14.03, 12.85) | -6.83 (-33.32, 19.54) |
|  | - | - | - | - | - | - | - | - | - | - | - | - | - | - | - | - | **Q** | - | - | - | - |
|  | 2.74 (-14.57, 19.63) | 7.04 (-10.52, 24.66) | 6.48 (-12.75, 25.16) | -5.31 (-24.53, 13.55) | 11.19 (-13.75, 36.02) | 17.87 (-1.44, 36.95) | 16.53 (-7.13, 40.02) | - | 14.83 (-5.67, 35.08) | 9.43 (-12.62, 31.22) | 10.39 (-14.89, 35.25) | 9.87 (-7.65, 26.98) | 9.78 (-8, 27.17) | 14.36 (-3.63, 32.32) | 8.92 (-8.42, 25.97) | 9.98 (-7.58, 27.2) | - | **R** | -0.11 (-25.72, 25.16) | -2.45 (-25.7, 20.64) | -8.55 (-41.7, 23.75) |
|  | **-8.73 (-16.2, -1.4)** | -4.3 (-12.5, 3.58) | -4.85 (-16.25, 6.18) | **-16.69 (-27.91, -5.3)** | -0.03 (-19.61, 19.09) | 6.51 (-4.86, 17.69) | 5.25 (-12.93, 22.8) | - | 3.47 (-10.04, 16.71) | -2 (-17.57, 13.63) | -0.95 (-20.48, 18.22) | -1.57 (-9.62, 6.19) | -1.65 (-10.23, 6.79) | 2.92 (-6.57, 12.49) | -2.52 (-9.68, 4.51) | -1.45 (-9.56, 6.56) | - | -11.4 (-29.93, 7.3) | **S** | -2.35 (-18.2, 13.49) | -8.43 (-37.47, 20.26) |
|  | **-9.91 (-13.4, -6.55)** | **-5.54 (-10.39, -0.72)** | -6.07 (-15.13, 2.89) | **-17.86 (-27.17, -8.45)** | -1.21 (-19.78, 16.9) | 5.29 (-4.01, 14.53) | 4.07 (-12.96, 20.5) | - | 2.2 (-9.59, 13.84) | -3.16 (-17.28, 10.77) | -2.11 (-20.62, 15.91) | -2.76 (-7.24, 1.62) | -2.89 (-7.91, 2.07) | 1.74 (-5.25, 8.73) | **-3.72 (-7, -0.53)** | -2.64 (-7.38, 2.16) | - | -12.61 (-29.87, 5.02) | -1.22 (-8.97, 6.69) | **T** | -6.25 (-33.14, 20.85) |
|  | **-17.57 (-27.15, -7.97)** | **-13.12 (-23.51, -2.78)** | **-13.68 (-24.84, -2.75)** | **-25.48 (-38.2, -12.58)** | -8.91 (-29.69, 11.33) | -2.38 (-15.39, 10.63) | -3.68 (-22.85, 15.2) | - | -5.41 (-18.78, 7.83) | -10.77 (-27.52, 5.88) | -9.82 (-30.33, 10.4) | **-10.41 (-20.38, -0.45)** | -10.51 (-21.02, 0.09) | -5.88 (-13.21, 1.42) | **-11.36 (-21.23, -1.57)** | **-10.3 (-20.29, -0.1)** | - | **-20.21 (-39.79, -0.81)** | -8.78 (-20.9, 3.33) | -7.65 (-17.76, 2.6) | **U** |

A: Physical rehabilitation; B: Body acupuncture; C: Electro-acupuncture; D: Massage; E: Proprioceptive Neuromuscular Facilitation; F: Body acupuncture plus extracorporeal shock wave treatment; G: Body acupuncture plus proprioceptive neuromuscular facilitation; H: Body acupuncture plus massage; I: Electro-acupuncture plus repetitive transcranial magnetic stimulation; J: Physical rehabilitation plus continuous theta burst stimulation; K: Physical rehabilitation plus intermittent theta burst stimulation; L: Physical rehabilitation plus repetitive transcranial magnetic stimulation; M: Physical rehabilitation plus extracorporeal shock wave treatment; N: Physical rehabilitation plus electro-acupuncture; O: Physical rehabilitation plus body acupuncture; P: Physical rehabilitation plus massage; Q: Physical rehabilitation plus repetitive transcranial magnetic stimulation plus continuous theta burst stimulation; R: Physical rehabilitation plus repetitive transcranial magnetic stimulation plus intermittent theta burst stimulation; S: Physical rehabilitation plus repetitive transcranial magnetic stimulation plus body acupuncture; T: Physical rehabilitation plus extracorporeal shock wave treatment plus body acupuncture; U: Physical rehabilitation plus repetitive transcranial magnetic stimulation plus electro-acupuncture; FMA-UE: The Fugl-Meyer Assessment-Upper Extremity scale; MBI: The Modified Barthel Index scale.

Bold values indicate statistically significant results (95% CI excluding zero).
