# Supplementary material for: Comparison of the effects of different physical stimulation therapies on reducing upper limb spastic paralysis and motor dysfunction in stroke survivors after stroke: a network meta-analysis of randomized controlled trials
Source: Front Neurol. 2025 Apr 15;16:1554583. doi: 10.3389/fneur.2025.1554583 (PMC12037403; doi:10.3389/fneur.2025.1554583)
Supplement: Supplementary file 2 [file Data_Sheet_2.zip › Supplementary Table.docx]

***Supplementary Table***

***Comparison of the effects of different physical stimulation therapies on reducing upper limb spastic paralysis and motor dysfunction in stroke survivors after stroke: A network meta-analysis of randomized controlled trials***

**Mingtong Bian^1,2^, Fuyan Chen^1,2,3*^, Huizhen Su^3^, Zhiying Li^1,2^, Xiaowei Sun^1,2^, Yang Liu^1,2^,**

**Jinyuan Shi^1,2^, Shuo Liu^1,2^, Ru Rong^1,2^**

^1^Department of Acupuncture, First Teaching Hospital of Tianjin University of Traditional Chinese Medicine, Tianjin, China

^2^National Clinical Research Center for Chinese Medicine Acupuncture and Moxibustion, Tianjin, China

^3^Qinghai Provincial Hospital of Traditional Chinese Medicine,Qinghai,China

- **Table S1** Intervention Parameters and Implementation Details 3
- **Table S2** The results of the closed-loop inconsistency test 9
- **Table S3** Estimated Probability-Based Treatment Ranking Table 10
- **Table S4** Predictive Probability-Based Treatment Ranking Table 11
- **Table S5** The summary of Adverse Effects 12
- **Table S6** The results of Evidence Assessment 13

**Supplementary Table S1:** **Intervention Parameters and Implementation Details.**

| **Author** | **Year** | **Country** | **Intervention Group** | | | | **Control Group** | | | | **Follow-up** |
| --- | --- | --- | --- | --- | --- | --- | --- | --- | --- | --- | --- |
|  |  |  | **Intervention Details** | **Key Parameters** | **Duration per treatment** | **Frequency** | **Intervention Details** | **Key Parameters** | **Duration per treatment** | **Frequency** |  |
| **Ai YX** | 2023 | China | PR+ESWT+BA | ESWT: 8Hz, Hand: 1.0-2.0 bar; Elbow and Shoulder: 2.0-3.0 bar | ESWT: NA | ESWT: 1/4day | PR | Physical therapy, Occupational therapy | 40 min | 1/day | NA |
|  |  |  | PR+BA | BA: Upper limb acupoints, Scalp acupoints | BA: 1-15 min | BA: 1/day |  |  |  |  |  |
| **Bao YH** | 2012 | China | PR+EA | - | 75 min | 1/day | PR | Motor rehabilitation training, Bobath hand grasp | 45 min | 1/day | NA |
|  |  |  | EA | 2Hz, Upper limb acupoints | 30 min | 1/day |  |  |  |  |  |
| **Barros G** | 2014 | Brazil | PR+rTMS | rTMS: 1 Hz, 90% RMT, M1 contralesional | rTMS: NA | 3/week | PR | Motor rehabilitation training | 30 min | 3/week | After 4 weeks |
| **Chen DY** | 2024 | China | PR+ESWT | ESWT: 8Hz, Upper limb: 3.0 bar | ESWT: NA | 2/week | PR | Motor rehabilitation training, Occupational therapy | 45 min | 6/week | NA |
| **Chen QF** | 2021 | China | PR+rTMS | rTMS: 1 Hz, 90% RMT, M1 contralesional | rTMS: 20 min | 5/week | PR | Motor rehabilitation training | 30 min | 5/week | NA |
| **Chen Y** | 2021 | China | PR+iTBS | iTBS: 80% AMT, ipsilesional lateral cerebellum | iTBS: NA | 1/day | PR | Motor rehabilitation training | 50 min | 1/day | NA |
| **Chen YJ** | 2019 | Taiwan, China | PR+iTBS | iTBS: 5 Hz, 80% AMT, psilesional primary motor cortex (M1) responsible for hand movement | iTBS: NA | 5/week | PR | Physical therapy, Occupational therapy | 90 min | 5/week | NA |
| **Chu GX** | 2009 | China | PR+EA | EA: 50-100Hz, Upper limb and lower limb acupoints | EA: 20 min | EA: 6/week | PR | Motor rehabilitation training | 30 min | 3/week | NA |
| **Dang YS** | 2020 | China | PR+BA | BA: Upper limb acupoints | BA: 20 min | 1/day | PR | Motor rehabilitation training, Occupational therapy | 40 min | 1/day | NA |
| **Gu YL** | 2018 | China | PR+M | M: Upper limb and back | M: 30 min | 1/day | PR | Motor rehabilitation training | 30 min | 1/day | NA |
| **Hao JB** | 2016 | China | PR+M | M: Upper limb | M: 30 min | 1/day | PR | Bobath therapy | 30 min | 1/day | NA |
| **Jiang YY** | 2023 | China | PR+rTMS+EA | rTMS: 1 Hz, 90% RMT, M1 contralesional; EA: Upper limb acupoints | rTMS: 20 min; EA: 30 min | 5/week | PR+EA | PR: Motor rehabilitation training, Occupational therapy | PR: NA | 5/week | NA |
| **Kuzu Ö** | 2021 | Turkey | PR+rTMS | rTMS: 1 Hz, 90% RMT, M1 contralesional | rTMS: 20 min | 1/week | PR | Physical therapy, Occupational therapy | 60 min | 1/week | After 4 weeks |
|  |  |  | PR+cTBS | cTBS: 50 Hz, 80% AMT, M1 contralesional | cTBS: 40 s | 1/week |  |  |  |  |  |
| **Lei JF** | 2024 | China | PR+rTMS+BA | rTMS: 1 Hz, 90% RMT, M1 contralesional; BA: Upper limb acupoints | rTMS: 20 min; BA: 30 min | 5/week | PR+BA | PR: Physical therapy, Motor rehabilitation training | PR: NA | 5/week | NA |
| **Lei M** | 2012 | China | PR+M | M: Upper limb | M: 30 min | 5/week | PR | Physical therapy, Occupational therapy | 45 min | 5/week | NA |
| **Li B** | 2021 | China | BA+M | M: Upper limb | M: 10 min | 6/week | BA | Upper limb and lower limb acupoints; Scalp acupoints | 20 min | 6/week | NA |
| **Li BJ** | 2017 | China | PR+BA | BA: Upper limb acupoints | BA: 20 min | 5/week | PR | Modified Constraint-Induced Movement Therapy | 6 h | 5/week | NA |
| **Li D** | 2021 | China | PR+rTMS+cTBS | PR: Physical therapy, Occupational therapy | PR: 40 min | 6/week | PR+rTMS | rTMS: 1 Hz, 80% RMT, M1 contralesional | rTMS: 20 min | 6/week | NA |
|  |  |  |  |  |  |  | PR+cTBS | cTBS: 50 Hz, 80% AMT, right cerebellum | cTBS: 80 s | 6/week |  |
| **Li ZW** | 2022 | China | PR+M | M: Upper limb | M: 30 min | 6/week | PR | Motor rehabilitation training | 30 min | 6/week | NA |
| **Lin FY** | 2018 | China | PR+BA | BA: Back acupoints | BA: 20 min | 5/week | PR | Motor rehabilitation training | NA | NA | NA |
| **Liu HJ** | 2023 | China | PR+BA | BA: Upper limb and lower limb acupoints; Scalp acupoints | BA: NA | 6/week | PR | Bobath therapy | 45 min | 6/week | NA |
| **Liu QQ** | 2021 | China | BA+PNF | BA: Upper limb acupoints; Scalp acupoints | BA: 30 min | 6/week | PNF | Upper limb | NA | 6/week | NA |
| **Liu SD** | 2023 | China | EA+rTMS | rTMS: 1 Hz, M1 contralesional | rTMS: 20 min | rTMS: 5/week | EA | 2～5 Hz, Upper limb acupoints | 30 min | 6/week | After 3 months |
| **Liu SH** | 2019 | China | PR+rTMS | rTMS: 1 Hz, 120% AMT, M1 contralesional | rTMS: 20 min | 6/week | PR | Physical therapy, Occupational therapy | NA | 6/week | NA |
| **Liu Y** | 2018 | China | PR+rTMS | rTMS: 1 Hz, 90% RMT, M1 contralesional | rTMS: 24 min | 5/week | PR | Motor rehabilitation training, Occupational therapy | 40 min | 5/week | NA |
| **Ma AF** | 2022 | China | PR+BA | BA: Upper limb acupoints | BA: 30 min | 5/week | PR | Motor rehabilitation training | 30 min | 5/week | NA |
| **Ma JY** | 2020 | China | BA+M | M: Upper limb | M: 10 min | 6/week | BA | Upper limb and lower limb acupoints; Scalp acupoints | 20 min | 6/week | NA |
| **Motamed V** | 2014 | Iran | PR+rTMS | rTMS: 1 Hz, 60-80% RMT, M1 contralesional | rTMS: 20 min | 3/week | PR | Motor rehabilitation training, Occupational therapy | 60 min | 3/week | NA |
| **Ni HH** | 2012 | China | PR+BA | BA: Upper limb acupoints | BA: 20 min | 1/2day | PR | Motor rehabilitation training, Occupational therapy | NA | 6/week | NA |
| **Qin Y** | 2023 | China | PR+rTMS | rTMS: 1 Hz, 90% RMT, M1 contralesional | rTMS: NA | 5/week | PR | Motor rehabilitation training | 40 min | 5/week | NA |
| **Shi J** | 2019 | China | BA+PNF | PNF: Upper limb | PNF: NA | 6/week | BA | Upper limb and lower limb acupoints; Scalp acupoints | 30 min | 6/week | NA |
| **Sun X** | 2023 | China | PR+ESWT+BA | ESWT: 8Hz, Upper limb: 1.5-2.0 bar | ESWT: NA | ESWT: 1/4day | PR+BA | PR: Physical therapy, Occupational therapy; BA: Upper limb acupoints, Scalp acupoints | PR: 1 h; BA: 30 min | 6/week | NA |
| **Sun YZ** | 2013 | China | PR+EA | EA: Upper limb acupoints | EA: 40 min | 6/week | PR | Brunnstrom therapy, Bobath therapy | NA | 6/week | NA |
| **Tong JY** | 2022 | China | PR+BA | BA: Upper limb acupoints | BA: NA | 1/day | PR | Bobath therapy | 40 min | 1/day | NA |
| **Wang CP** | 2014 | Taiwan, China | PR+rTMS+iTBS | rTMS: 1 Hz,90% RMT, M1 contralesional; iTBS: 80% AMT, ipsilesional M1 | rTMS: 10 min; iTBS: 190 s | 5/week | PR | Occupational therapy | 60 min | 5/week | After 3 months |
| **Wang J** | 2018 | China | PR+BA | PR: Bobath therapy | PR: NA | 1/day | BA | Upper limb acupoints | 20 min | 1/day | NA |
| **Wei CB** | 2021 | China | PR+ESWT+BA | - | - | - | PR | Motor rehabilitation training | 30 min | 2/day | NA |
|  |  |  | PR+ESWT | ESWT: 5Hz, Upper limb: 2.0 bar | ESWT: NA | ESWT: 1/week |  |  |  |  |  |
|  |  |  | PR+BA | BA: Upper limb acupoints | BA: 15 min | BA: 6/week |  |  |  |  |  |
| **Wen DG** | 2020 | China | PR+M | - | 80 min | 5/week | PR | Mirror therapy | 40 min | 5/week | NA |
|  |  |  |  |  |  |  | M | Upper limb | 40 min | 5/week |  |
| **Xie WX** | 2023 | China | PR+rTMS+BA | rTMS: 1 Hz, 80% RMT, M1 contralesional; BA: Upper limb and lower limb acupoints; Scalp acupoints | rTMS: 20 min; BA: 30 min | 5/week | PR+BA | PR: Physical therapy, Occupational therapy | PR: NA | 5/week | NA |
| **Xu SF** | 2016 | China | PR+BA | BA: Upper limb and lower limb acupoints; Scalp acupoints | BA: 30 min | 5/week | PR | Motor rehabilitation training | NA | 5/week | NA |
| **Xu YL** | 2010 | China | BA | Upper limb acupoints | 30 min | 5/week | PR | Bobath therapy | 1-2 h | 5/week | NA |
| **Yang NY** | 2017 | China | PR+rTMS | rTMS: 1 Hz, 90% RMT, P5 contralesional | rTMS: NA | 5/week | PR | Physical therapy, Occupational therapy | 45 min | 5/week | After 4 weeks |
| **Yang X** | 2021 | China | PR+rTMS | rTMS: 1 Hz, 90% RMT, M1 contralesional | rTMS: 24 min | 5/week | PR | Motor rehabilitation training | 30-40 min | 5/week | NA |
| **Zhang L** | 2015 | China | PR+BA | BA: Upper limb acupoints | BA: 20 min | 1/day | PR | Bobath therapy | NA | NA | NA |
| **Zhang QF** | 2021 | China | PR+M | M: Upper limb | M: 15 min | 1/day | PR | Motor rehabilitation training | 40 min | 1/day | NA |
| **Zhang X** | 2021 | China | PR+ESWT+BA | ESWT: 8Hz, Upper limb: 2.0-2.5 bar | ESWT: NA | ESWT: 1/week | PR+BA | PR: Physical therapy, Occupational therapy; BA: Upper limb acupoints | PR: 120 min; BA: 30 min | 5/week | After 3 months |
| **Zhao J** | 2021 | China | PR+rTMS | rTMS: 1 Hz, 80% RMT, M1 contralesional | rTMS: NA | 6/week | PR | Motor rehabilitation training | ＞3 h | 6/week | After 2 weeks |
| **Zhao JY** | 2021 | China | BA+ ESWT | ESWT: 8Hz, Upper limb: 1.5-2.0 bar | ESWT: 25 min | ESWT: 2/week | BA | Upper limb acupoints | 30 min | 6/week | NA |
| **Zhou P** | 2019 | China | PR+BA | PR: Motor rehabilitation training, Bobath therapy | PR: 45 min | 5/week | BA | Upper limb and lower limb acupoints | NA | 5/week | NA |

PR: Physical rehabilitation; BA: Body acupuncture; EA: Electro-acupuncture; M: Massage; PNF: Proprioceptive Neuromuscular Facilitation; ESWT: Extracorporeal shock wave treatment; rTMS: repetitive Transcranial Magnetic Stimulation; cTBS: continuous Theta Burst Stimulation; iTBS: intermittent Theta Burst Stimulation.

**Supplementary Table S2: The results of the closed-loop inconsistency test.**

| **Loop** | **IF** | **seIF** | **Z_value** | **P_value** | **95%CI** | **Loop_Heterog_tau2** |
| --- | --- | --- | --- | --- | --- | --- |
| **FMA-UE** | | | | | | |
| **A-C-M** | 7.490 | 5.401 | 1.387 | 0.166 | **(0.00,18.08)** | 0.000 |
| **A-I-K** | 7.464 | 10.931 | 0.683 | 0.495 | **(0.00,28.89)** | 11.165 |
| **A-L-S** | 5.87 | 1.628 | 3.605 | 0.000 | **(2.68,9.06)** | 0.000 |
| **A-N-S** | 3.827 | 3.346 | 1.144 | 0.253 | **(0.00,10.39)** | 11.574 |
| **A-B-N** | 3.255 | 3.799 | 0.857 | 0.392 | **(0.00,10.70)** | 11.701 |
| **A-D-O** | 1.772 | 6.476 | 0.274 | 0.784 | **(0.00,14.46)** | 1.633 |
| **L-N-S** | 1.412 | 1.412 | 0.820 | 0.820 | **(0.00,4.79)** | 0.000 |
| **A-L-N** | 0.132 | 5.032 | 0.026 | 0.979 | **(0.00,10.00)** | 14.349 |
| **MBI** | | | | | | |
| **A-D-P** | 35.799 | 4.526 | 7.909 | 0.000 | **(26.93,44.67)** | 0.000 |
| **M-O-T** | 6.492 | 8.089 | 0.802 | 0.422 | **(0.00,22.35)** | 0.000 |
| **A-M-T** | 5.872 | 6.461 | 0.909 | 0.363 | **(0.00,18.53)** | 0.000 |
| **A-M-O** | 5.366 | 8.099 | 0.663 | 0.508 | **(0.00,21.24)** | 17.862 |
| **A-O-T** | 4.565 | 4.273 | 1.068 | 0.285 | **(0.00,12.94)** | 9.974 |
| **J-L-Q** | - | - | - | - | **-** | 0.000 |

*** Note: Loop J-L-Q is formed only by multi-arm trial(s) - Consistent by definition

A: Physical rehabilitation; B: Body acupuncture; C: Electro-acupuncture; D: Massage; E: Proprioceptive Neuromuscular Facilitation; F: Body acupuncture plus extracorporeal shock wave treatment; G: Body acupuncture plus proprioceptive neuromuscular facilitation; H: Body acupuncture plus massage; I: Electro-acupuncture plus repetitive transcranial magnetic stimulation; J: Physical rehabilitation plus continuous theta burst stimulation; K: Physical rehabilitation plus intermittent theta burst stimulation; L: Physical rehabilitation plus repetitive transcranial magnetic stimulation; M: Physical rehabilitation plus extracorporeal shock wave treatment; N: Physical rehabilitation plus electro-acupuncture; O: Physical rehabilitation plus body acupuncture; P: Physical rehabilitation plus massage; Q: Physical rehabilitation plus repetitive transcranial magnetic stimulation plus continuous theta burst stimulation; R: Physical rehabilitation plus repetitive transcranial magnetic stimulation plus intermittent theta burst stimulation; S: Physical rehabilitation plus repetitive transcranial magnetic stimulation plus body acupuncture; T: Physical rehabilitation plus extracorporeal shock wave treatment plus body acupuncture; U: Physical rehabilitation plus repetitive transcranial magnetic stimulation plus electro-acupuncture; FMA-UE: The Fugl-Meyer Assessment-Upper Extremity scale; MBI: The Modified Barthel Index scale.

**Supplementary Table S3: Estimated Probability-Based Treatment Ranking Table.**

| **Treatment** | **FMA-UE** | | | **MBI** | | |
| --- | --- | --- | --- | --- | --- | --- |
|  | **SUCRA** | **Pr Best** | **MeanRank** | **SUCRA** | **Pr Best** | **MeanRank** |
| **A** | 13.2 | 0.0 | 16.6 | 20.8 | 0.0 | 14.5 |
| **B** | 29.3 | 0.0 | 13.7 | 23.8 | 0.0 | 14.0 |
| **C** | 30.3 | 0.0 | 13.5 | - | - | - |
| **D** | 2.9 | 0.0 | 18.5 | 34.8 | 0.3 | 12.1 |
| **E** | 52.7 | 2.2 | 9.5 | 34.3 | 2.2 | 12.2 |
| **F** | 84.6 | 16.8 | 3.8 | 61.3 | 10.4 | 7.6 |
| **G** | 74.8 | 22.2 | 5.5 | 55.7 | 7.7 | 8.5 |
| **H** | - | - | - | 51.1 | 2.3 | 9.3 |
| **I** | 72.5 | 8.1 | 6.0 | - | - | - |
| **J** | 47.9 | 3.5 | 10.4 | 51.6 | 3.0 | 9.2 |
| **K** | 50.6 | 8.5 | 9.9 | 9.9 | 0.0 | 16.3 |
| **L** | 48.2 | 0.0 | 10.3 | 42.9 | 0.0 | 10.7 |
| **M** | 47.4 | 0.0 | 10.5 | 57.2 | 1.0 | 8.3 |
| **N** | 73.1 | 0.2 | 5.8 | 27.1 | 0.0 | 13.4 |
| **O** | 40.2 | 0.0 | 11.8 | 51.3 | 0.0 | 9.3 |
| **P** | 49.1 | 0.0 | 10.2 | 80.6 | 8.8 | 4.3 |
| **Q** | - | - | - | 79.0 | 27.0 | 4.6 |
| **R** | 17.5 | 0.6 | 15.8 | - | - | - |
| **S** | 57.3 | 0.5 | 8.7 | **83.1** | 20.1 | 3.9 |
| **T** | 67.3 | 0.1 | 6.9 | 78.1 | 6.4 | 4.7 |
| **U** | **91.1** | 37.3 | 2.6 | 57.4 | 10.7 | 8.2 |

A: Physical rehabilitation; B: Body acupuncture; C: Electro-acupuncture; D: Massage; E: Proprioceptive Neuromuscular Facilitation; F: Body acupuncture plus extracorporeal shock wave treatment; G: Body acupuncture plus proprioceptive neuromuscular facilitation; H: Body acupuncture plus massage; I: Electro-acupuncture plus repetitive transcranial magnetic stimulation; J: Physical rehabilitation plus continuous theta burst stimulation; K: Physical rehabilitation plus intermittent theta burst stimulation; L: Physical rehabilitation plus repetitive transcranial magnetic stimulation; M: Physical rehabilitation plus extracorporeal shock wave treatment; N: Physical rehabilitation plus electro-acupuncture; O: Physical rehabilitation plus body acupuncture; P: Physical rehabilitation plus massage; Q: Physical rehabilitation plus repetitive transcranial magnetic stimulation plus continuous theta burst stimulation; R: Physical rehabilitation plus repetitive transcranial magnetic stimulation plus intermittent theta burst stimulation; S: Physical rehabilitation plus repetitive transcranial magnetic stimulation plus body acupuncture; T: Physical rehabilitation plus extracorporeal shock wave treatment plus body acupuncture; U: Physical rehabilitation plus repetitive transcranial magnetic stimulation plus electro-acupuncture; FMA-UE: The Fugl-Meyer Assessment-Upper Extremity scale; MBI: The Modified Barthel Index scale.

**Supplementary Table S4: Predictive Probability-Based Treatment Ranking Table.**

| **Treatment** | **FMA-UE** | | | **MBI** | | |
| --- | --- | --- | --- | --- | --- | --- |
|  | **SUCRA** | **Pr Best** | **MeanRank** | **SUCRA** | **Pr Best** | **MeanRank** |
| **A** | 14.8 | 0.0 | 16.3 | 25.6 | 0.0 | 13.7 |
| **B** | 32.8 | 0.0 | 13.1 | 27.6 | 0.0 | 13.3 |
| **C** | 32.0 | 0.0 | 13.2 | - | - | - |
| **D** | 3.5 | 0.0 | 18.4 | 36.3 | 0.6 | 11.8 |
| **E** | 53.1 | 3.6 | 9.4 | 35.6 | 2.6 | 12.0 |
| **F** | 83.1 | 16.8 | 4.0 | 59.8 | 9.5 | 7.8 |
| **G** | 74.0 | 21.0 | 5.7 | 54.8 | 7.7 | 8.7 |
| **H** | - | - | - | 50.5 | 3.0 | 9.4 |
| **I** | 70.9 | 8.8 | 6.2 | - | - | - |
| **J** | 47.3 | 3.2 | 10.5 | 51.8 | 3.6 | 9.2 |
| **K** | 50.6 | 8.5 | 9.9 | 13.0 | 0.1 | 15.8 |
| **L** | 48.2 | 0.0 | 10.3 | 44.5 | 0.2 | 10.4 |
| **M** | 47.7 | 0.1 | 10.4 | 56.5 | 2.2 | 8.4 |
| **N** | 71.3 | 1.3 | 6.2 | 30.0 | 0.2 | 12.9 |
| **O** | 42.4 | 0.0 | 11.4 | 51.2 | 0.3 | 9.3 |
| **P** | 49.1 | 0.1 | 10.2 | 76.6 | 10.0 | 5.0 |
| **Q** | - | - | - | 76.4 | 23.3 | 5.0 |
| **R** | 18.1 | 0.5 | 15.7 | - | - | - |
| **S** | 56.8 | 0.9 | 8.8 | **78.8** | 18.2 | 4.6 |
| **T** | 64.7 | 0.4 | 7.4 | 74.3 | 8.9 | 5.4 |
| **U** | **89.4** | 34.7 | 2.9 | 56.8 | 9.7 | 8.4 |

A: Physical rehabilitation; B: Body acupuncture; C: Electro-acupuncture; D: Massage; E: Proprioceptive Neuromuscular Facilitation; F: Body acupuncture plus extracorporeal shock wave treatment; G: Body acupuncture plus proprioceptive neuromuscular facilitation; H: Body acupuncture plus massage; I: Electro-acupuncture plus repetitive transcranial magnetic stimulation; J: Physical rehabilitation plus continuous theta burst stimulation; K: Physical rehabilitation plus intermittent theta burst stimulation; L: Physical rehabilitation plus repetitive transcranial magnetic stimulation; M: Physical rehabilitation plus extracorporeal shock wave treatment; N: Physical rehabilitation plus electro-acupuncture; O: Physical rehabilitation plus body acupuncture; P: Physical rehabilitation plus massage; Q: Physical rehabilitation plus repetitive transcranial magnetic stimulation plus continuous theta burst stimulation; R: Physical rehabilitation plus repetitive transcranial magnetic stimulation plus intermittent theta burst stimulation; S: Physical rehabilitation plus repetitive transcranial magnetic stimulation plus body acupuncture; T: Physical rehabilitation plus extracorporeal shock wave treatment plus body acupuncture; U: Physical rehabilitation plus repetitive transcranial magnetic stimulation plus electro-acupuncture; FMA-UE: The Fugl-Meyer Assessment-Upper Extremity scale; MBI: The Modified Barthel Index scale.

**Supplementary Table S5: The summary of Adverse Effects.**

| **Study** | **Adverse Effects** |
| --- | --- |
| **Bao YH 2012** | In the electro-acupuncture group, subcutaneous bruising was occasionally observed following treatment. This was reported to be absorbed and resolved within two weeks, with no other adverse reactions documented. |
| **Chen Y 2021** | No adverse effects. |
| **Kuzu Ö 2021** | No adverse effects. |
| **Jiang YY 2023** | One patient in the observation group reported mild discomfort at the rTMS stimulation site during the course of treatment, which subsequently abated. |
| **Liu HJ 2023** | No adverse effects. |
| **Liu SD 2023** | No adverse effects. |
| **Ma AF 2022** | No adverse effects. |
| **Motamed V 2014** | No adverse effects. |
| **Sun X 2023** | The primary adverse reactions observed in patients were musculoskeletal discomfort at the treatment site, which was generally well-tolerated. No evidence of petechiae or ecchymosis was discernible, and no other discomfort was reported. |
| **Wang CP 2014** | No adverse effects. |
| **Wei CB 2021** | No adverse effects. |
| **Xie WX 2023** | Only a small number of patients reported discomfort at the stimulation site during the initial rTMS treatment, and no other adverse reactions were observed. |
| **Xu SF 2016** | No adverse effects. |
| **Yang NY 2017** | No adverse effects. |
| **Yang X 2021** | No adverse effects. |
| **Zhang X 2021** | No adverse effects. |

**Supplementary Table S6: The results of Evidence Assessment.**

| **Comparison** | **Risk of bias** | **Inconsistency** | **Indirectness** | **Imprecision** | **Publication Bias** | **GRADE** |
| --- | --- | --- | --- | --- | --- | --- |
| **FMA-UE** | | | | | | |
| **B-A** | Serious | Not serious | Not serious | Not serious | Not serious | MODERATE |
| **C-A** | Serious | Not serious | Not serious | Very serious | Not serious | VERY LOW |
| **D-A** | Serious | Not serious | Not serious | Very serious | Not serious | VERY LOW |
| **J-A** | Serious | Not serious | Not serious | Very serious | Not serious | VERY LOW |
| **K-A** | Serious | Not serious | Not serious | Very serious | Not serious | VERY LOW |
| **L-A** | Serious | Serious | Not serious | Not serious | Not serious | LOW |
| **M-A** | Serious | Not serious | Not serious | Not serious | Not serious | MODERATE |
| **N-A** | Serious | Serious | Not serious | Not serious | Not serious | LOW |
| **O-A** | Serious | Serious | Not serious | Not serious | Not serious | LOW |
| **P-A** | Serious | Not serious | Not serious | Not serious | Not serious | MODERATE |
| **R-A** | Serious | Not serious | Not serious | Very serious | Not serious | VERY LOW |
| **T-A** | Serious | Serious | Not serious | Not serious | Not serious | LOW |
| **F-B** | Serious | Not serious | Not serious | Serious | Not serious | LOW |
| **G-B** | Serious | Not serious | Not serious | Very serious | Not serious | VERY LOW |
| **O-B** | Serious | Serious | Not serious | Very serious | Not serious | VERY LOW |
| **I-C** | Serious | Not serious | Not serious | Not serious | Not serious | MODERATE |
| **N-C** | Serious | Not serious | Not serious | Very serious | Not serious | VERY LOW |
| **P-D** | Serious | Not serious | Not serious | Serious | Not serious | LOW |
| **G-E** | Serious | Not serious | Not serious | Very serious | Not serious | VERY LOW |
| **L-J** | Serious | Not serious | Not serious | Very serious | Not serious | VERY LOW |
| **O-M** | Serious | Not serious | Not serious | Very serious | Not serious | VERY LOW |
| **T-M** | Serious | Not serious | Not serious | Serious | Not serious | LOW |
| **U-N** | Serious | Not serious | Not serious | Very serious | Not serious | VERY LOW |
| **S-O** | Serious | Not serious | Not serious | Very serious | Not serious | VERY LOW |
| **T-O** | Serious | Not serious | Not serious | Serious | Not serious | LOW |
| **MBI** | | | | | | |
| **D-A** | Serious | Not serious | Not serious | Very serious | Not serious | VERY LOW |
| **K-A** | Serious | Not serious | Not serious | Very serious | Not serious | VERY LOW |
| **L-A** | Serious | Serious | Not serious | Not serious | Not serious | LOW |
| **M-A** | Serious | Not serious | Not serious | Very serious | Not serious | VERY LOW |
| **N-A** | Serious | Not serious | Not serious | Very serious | Not serious | VERY LOW |
| **O-A** | Serious | Serious | Not serious | Not serious | Not serious | LOW |
| **P-A** | Serious | Serious | Not serious | Not serious | Not serious | LOW |
| **T-A** | Serious | Serious | Not serious | Not serious | Not serious | LOW |
| **F-B** | Serious | Not serious | Not serious | Very serious | Not serious | VERY LOW |
| **G-B** | Serious | Not serious | Not serious | Very serious | Not serious | VERY LOW |
| **H-B** | Serious | Not serious | Not serious | Very serious | Not serious | VERY LOW |
| **O-B** | Serious | Not serious | Not serious | Very serious | Not serious | VERY LOW |
| **P-D** | Serious | Not serious | Not serious | Very serious | Not serious | VERY LOW |
| **G-E** | Serious | Not serious | Not serious | Very serious | Not serious | VERY LOW |
| **L-J** | Serious | Not serious | Not serious | Very serious | Not serious | VERY LOW |
| **Q-J** | Serious | Not serious | Not serious | Very serious | Not serious | VERY LOW |
| **Q-L** | Serious | Not serious | Not serious | Very serious | Not serious | VERY LOW |
| **O-M** | Serious | Not serious | Not serious | Very serious | Not serious | VERY LOW |
| **T-M** | Serious | Not serious | Not serious | Very serious | Not serious | VERY LOW |
| **U-N** | Serious | Not serious | Not serious | Very serious | Not serious | VERY LOW |
| **S-O** | Serious | Not serious | Not serious | Very serious | Not serious | VERY LOW |
| **T-O** | Serious | Not serious | Not serious | Very serious | Not serious | VERY LOW |

A: Physical rehabilitation; B: Body acupuncture; C: Electro-acupuncture; D: Massage; E: Proprioceptive Neuromuscular Facilitation; F: Body acupuncture plus extracorporeal shock wave treatment; G: Body acupuncture plus proprioceptive neuromuscular facilitation; H: Body acupuncture plus massage; I: Electro-acupuncture plus repetitive transcranial magnetic stimulation; J: Physical rehabilitation plus continuous theta burst stimulation; K: Physical rehabilitation plus intermittent theta burst stimulation; L: Physical rehabilitation plus repetitive transcranial magnetic stimulation; M: Physical rehabilitation plus extracorporeal shock wave treatment; N: Physical rehabilitation plus electro-acupuncture; O: Physical rehabilitation plus body acupuncture; P: Physical rehabilitation plus massage; Q: Physical rehabilitation plus repetitive transcranial magnetic stimulation plus continuous theta burst stimulation; R: Physical rehabilitation plus repetitive transcranial magnetic stimulation plus intermittent theta burst stimulation; S: Physical rehabilitation plus repetitive transcranial magnetic stimulation plus body acupuncture; T: Physical rehabilitation plus extracorporeal shock wave treatment plus body acupuncture; U: Physical rehabilitation plus repetitive transcranial magnetic stimulation plus electro-acupuncture; FMA-UE: The Fugl-Meyer Assessment-Upper Extremity scale; MBI: The Modified Barthel Index scale.
