# Supplementary material for: Comparison of the effects of different physical stimulation therapies on reducing upper limb spastic paralysis and motor dysfunction in stroke survivors after stroke: a network meta-analysis of randomized controlled trials
Source: Front Neurol. 2025 Apr 15;16:1554583. doi: 10.3389/fneur.2025.1554583 (PMC12037403; doi:10.3389/fneur.2025.1554583)
Supplement: Supplementary file 2 [file Data_Sheet_2.zip › supplementary Appendix S2.docx]

**The search strategy for the PubMed database**

| **No** | **Search History** |
| --- | --- |
| #1 | "Stroke"[Mesh] OR "Brain Ischemia"[Mesh] OR "Cerebral Hemorrhage"[Mesh] |
| #2 | (((“Strokes” [Title/Abstract]) OR (Cerebrovascular Accident [Title/Abstract])) OR (Cerebrovascular Accidents [Title/Abstract])) OR (Cerebrovascular Accidents, Acute [Title/Abstract])) OR (Acute Cerebrovascular Accident [Title/Abstract])) OR Acute Cerebrovascular Accidents [Title/Abstract])) OR (Cerebrovascular Apoplexy [Title/Abstract])) OR (Apoplexy, Cerebrovascular [Title/Abstract])) OR (Vascular Accident, Brain [Title/Abstract])) OR (Brain Vascular Accidents [Title/Abstract])) OR (Cerebrovascular Stroke [Title/Abstract])) OR (Apoplexy [Title/Abstract])) OR (Cerebral Stroke [Title/Abstract])) OR (Acute Stroke [Title/Abstract]))) |
| #3 | (#1 OR #2) |
| #4 | "Acupuncture"[Mesh] OR "Acupuncture Therapy"[Mesh] OR "Transcranial Magnetic Stimulation"[Mesh] OR "Massage"[Mesh] |
| #5 | ((((((((((((((Electroacupuncture [Title/Abstract]) OR (Manual needle [Title/Abstract])) OR (body needle [Title/Abstract])) OR (needle [Title/Abstract])) OR (acupuncture treatment [Title/Abstract])) OR (Low-Frequency Pulsed Eectrical Simulation [Title/Abstract])) OR (Low-Frequency Stimulation [Title/Abstract])) OR (Magnetic Stimulations, Transcranial [Title/Abstract])) OR (Transcranial Magnetic Stimulations [Title/Abstract])) OR (Stimulations, Transcranial Magnetic [Title/Abstract])) OR (Transcranial Magnetic Stimulation, Repetitive [Title/Abstract])) OR (Zone Therapy [Title/Abstract])) OR (Zone Therapies [Title/Abstract])) OR (Massage Therapy [Title/Abstract])) OR (Therapies, Massage [Title/Abstract]) |
| #6 | (#4 OR #5) |
| #7 | "Hemiplegia, Spastic"[Mesh] |
| #8 | “Hemiplegias, Spastic” [Title/Abstract]) OR (Spastic Hemiplegia [Title/Abstract])) OR (Spastic Hemiplegias [Title/Abstract])) OR (Spastic Hemiplegias [Title/Abstract])) OR (Muscle Spasticity [Title/Abstract])) OR (Spasticity, Muscle [Title/Abstract])) OR (Spastic [Title/Abstract])) OR (Clasp-Knife Spasticity [Title/Abstract])) OR (Hypertonicity [Title/Abstract])) OR (Muscle Hypertonia [Title/Abstract])) OR (Hypertonias, Muscle [Title/Abstract])) OR (Increased, Muscle Tone [Title/Abstract]) OR (Hypermyotonia [Title/Abstract]))) |
| #9 | (#7 OR #8) |
| #10 | "Upper Extremity"[Mesh] |
| #11 | (((((((Extremities, Upper [Title/Abstract]) OR (Upper Extremities [Title/Abstract])) OR (Extremity, Upper [Title/Abstract])) OR (Membrum superius [Title/Abstract])) OR (Upper Limb [Title/Abstract])) OR (Limbs, Upper [Title/Abstract])) OR (Limb, Upper [Title/Abstract])) OR (Upper Limbs [Title/Abstract]) |
| #12 | (#10 OR #11) |
| #10 | (#3 AND #6 AND #9 AND #12) |

**The search strategy for the Web of Science database**

| **No** | **Search History** |
| --- | --- |
| #1 | TS=(Stroke OR Strokes OR Cerebrovascular Accident OR Cerebrovascular Accidents OR CVA (Cerebrovascular Accident) OR CVAs (Cerebrovascular Accident) OR Cerebrovascular Apoplexy OR Apoplexy, Cerebrovascular OR Vascular Accident, Brain OR Brain Vascular Accident OR Brain Vascular Accidents OR Vascular Accidents, Brain OR Cerebrovascular Stroke OR Cerebrovascular Strokes OR Stroke, Cerebrovascular OR Strokes, Cerebrovascular OR Apoplexy OR Cerebral Stroke OR Cerebral Strokes OR Stroke, Cerebral OR Strokes, Cerebral OR Stroke, Acute OR Acute Stroke OR Acute Strokes OR Strokes, Acute OR Cerebrovascular Accident, Acute OR Acute Cerebrovascular Accident OR Acute Cerebrovascular Accidents OR Cerebrovascular Accidents, Acute |
| #2 | TS=(Acupuncture OR Acupuncture Therapy OR Electroacupuncture OR Manual needle OR body needle OR needle OR acupuncture treatment OR Low-Frequency Pulsed Eectrical Simulation OR Low-Frequency Stimulation OR Transcranial Magnetic Stimulation OR Magnetic Stimulations, Transcranial OR Transcranial Magnetic Stimulations OR Stimulations, Transcranial Magnetic OR Transcranial Magnetic Stimulation, Repetitive OR Massage OR Zone Therapy OR Zone Therapies OR Massage Therapy OR Therapies, Massage) |
| #3 | TS=(Hemiplegia, Spastic OR Hemiplegias, Spastic OR Spastic Hemiplegia OR Spastic Hemiplegias OR Spasticity OR Muscle Spasticity OR Spasticity, Muscle OR Spastic OR Clasp-Knife Spasticity OR Clasp Knife Spasticity OR Spasticity, Clasp-Knife OR Hypertonicity OR Muscle Hypertonia OR Hypertonia, Muscle OR Hypertonias, Muscle OR Muscle Hypertonias OR Muscle Tone Increased OR Increased, Muscle Tone OR Tone Increased, Muscle OR Muscular Hypertonicity OR Hypertonicities, Muscular OR Hypertonicity, Muscular OR Muscular Hypertonicities OR Hypermyotonia OR Hypermyotonias) |
| #4 | TS=(Upper Extremity OR Extremities, Upper OR Upper Extremities OR Extremity, Upper OR Membrum superius OR Upper Limb OR Limbs, Upper OR Limb, Upper OR Upper Limbs) |
| #5 | (#1 AND #2 AND #3 AND #4) |

**The search strategy for Embase database**

| **No** | **Search History** |
| --- | --- |
| #1 | Stroke |
| #2 | 'Strokes':ab,ti,kw OR 'Cerebrovascular Accident':ab,ti,kw OR 'Cerebrovascular Accidents':ab,ti,kw OR 'Cerebrovascular Accident':ab,ti,kw OR 'Cerebrovascular Accident':ab,ti,kw OR 'Cerebrovascular Apoplexy':ab,ti,kw OR 'Apoplexy, Cerebrovascular':ab,ti,kw OR 'Vascular Accident, Brain':ab,ti,kw OR 'Brain Vascular Accident':ab,ti,kw OR 'Brain Vascular Accidents':ab,ti,kw OR 'Vascular Accidents, Brain':ab,ti,kw OR 'Cerebrovascular Stroke':ab,ti,kw OR 'Cerebrovascular Strokes':ab,ti,kw OR 'Stroke, Cerebrovascular':ab,ti,kw OR 'Strokes, Cerebrovascular':ab,ti,kw OR 'Apoplexy':ab,ti,kw OR 'Cerebral Stroke':ab,ti,kw OR 'Cerebral Strokes':ab,ti,kw OR 'Stroke, Cerebral':ab,ti,kw OR 'Strokes, Cerebral':ab,ti,kw OR 'Stroke, Acute':ab,ti,kw OR 'Acute Stroke':ab,ti,kw OR 'Acute Strokes':ab,ti,kw OR 'Strokes, Acute':ab,ti,kw OR 'Cerebrovascular Accident, Acute':ab,ti,kw OR 'Acute Cerebrovascular Accident':ab,ti,kw OR 'Acute Cerebrovascular Accidents':ab,ti,kw OR 'Cerebrovascular Accidents, Acute':ab,ti,kw |
| #3 | (#1 OR #2) |
| #4 | 'Muscle Spasticity' |
| #5 | 'Hemiplegia, Spastic':ab,ti,kw OR 'Hemiplegias, Spastic':ab,ti,kw OR 'Spastic Hemiplegia':ab,ti,kw OR 'Spastic Hemiplegias':ab,ti,kw OR 'Spasticity':ab,ti,kw OR 'Spasticity, Muscle':ab,ti,kw OR 'Spastic':ab,ti,kw OR 'Clasp-Knife Spasticity':ab,ti,kw OR 'Clasp Knife Spasticity':ab,ti,kw OR 'Spasticity, Clasp-Knife':ab,ti,kw OR 'Hypertonicity':ab,ti,kw OR 'Muscle Hypertonia':ab,ti,kw OR 'Hypertonia, Muscle':ab,ti,kw OR 'Hypertonias, Muscle':ab,ti,kw OR 'Muscle Hypertonias':ab,ti,kw OR 'Muscle Tone Increased':ab,ti,kw OR 'Increased, Muscle Tone':ab,ti,kw OR 'Tone Increased, Muscle':ab,ti,kw OR 'Muscular Hypertonicity':ab,ti,kw OR 'Hypertonicities, Muscular':ab,ti,kw OR 'Hypertonicity, Muscular':ab,ti,kw OR 'Muscular Hypertonicities':ab,ti,kw OR 'Hypermyotonia':ab,ti,kw OR 'Hypermyotonias':ab,ti,kw |
| #6 | (#4 OR #5) |
| #7 | 'Upper Extremity' |
| #8 | 'Extremities, Upper':ab,ti,kw OR 'Upper Extremities':ab,ti,kw OR 'Extremity, Upper':ab,ti,kw OR 'Membrum superius':ab,ti,kw OR 'Upper Limb':ab,ti,kw OR 'Limbs, Upper':ab,ti,kw OR 'Limb, Upper':ab,ti,kw OR 'Upper Limbs':ab,ti,kw |
| #9 | (#7 OR #8) |
| #10 | Acupuncture |
| #11 | 'Electroacupuncture':ab,ti,kw OR 'Manual needle':ab,ti,kw OR 'body needle':ab,ti,kw OR 'Needle':ab,ti,kw OR 'acupuncture treatment':ab,ti,kw |
| #12 | (#10 OR #11) |
| #13 | Massage |
| #14 | 'Zone Therapy':ab,ti,kw OR 'Therapies, Zone':ab,ti,kw OR 'Zone Therapies':ab,ti,kw OR 'Therapy, Zone':ab,ti,kw OR 'Massage Therapy':ab,ti,kw OR 'Massage Therapies':ab,ti,kw OR 'Therapies, Massage':ab,ti,kw OR 'Therapy, Massage':ab,ti,kw |
| #15 | (#13 OR #14) |
| #16 | 'Transcranial Magnetic Stimulation' |
| #17 | 'Magnetic Stimulations, Transcranial':ab,ti,kw OR 'Magnetic Stimulation, Transcranial':ab,ti,kw OR 'Stimulations, Transcranial Magnetic':ab,ti,kw OR 'Stimulation, Transcranial Magnetic':ab,ti,kw OR 'Transcranial Magnetic Stimulations':ab,ti,kw OR 'Transcranial Magnetic Stimulation, Paired Pulse':ab,ti,kw OR 'Transcranial Magnetic Stimulation, Repetitive':ab,ti,kw OR 'Transcranial Magnetic Stimulation, Single Pulse':ab,ti,kw |
| #18 | (#16 OR #17) |
| #19 | 'Low-Frequency Stimulation':ab,ti,kw OR 'Low-Frequency Pulsed Eectrical Simulation':ab,ti,kw |
| #20 | (#12 OR #15 OR #18 OR #19) |
| #21 | (#3 AND #6 AND #9 AND #20) |

**The search strategy for the Cochrane Library database**

| **No** | **Search History** |
| --- | --- |
| #1 | Stroke |
| #2 | (Strokes):ab,ti,kw OR (Cerebrovascular Accident):ab,ti,kw OR (Cerebrovascular Accidents):ab,ti,kw OR (CVA (Cerebrovascular Accident)):ab,ti,kw OR (CVAs (Cerebrovascular Accident)):ab,ti,kw OR (Cerebrovascular Apoplexy):ab,ti,kw OR (Apoplexy, Cerebrovascular):ab,ti,kw OR (Vascular Accident, Brain):ab,ti,kw OR (Brain Vascular Accident):ab,ti,kw OR (Brain Vascular Accidents):ab,ti,kw OR (Vascular Accidents, Brain):ab,ti,kw OR (Cerebrovascular Stroke):ab,ti,kw OR (Cerebrovascular Strokes):ab,ti,kw OR (Stroke, Cerebrovascular):ab,ti,kw OR (Strokes, Cerebrovascular):ab,ti,kw OR (Apoplexy):ab,ti,kw OR (Cerebral Stroke):ab,ti,kw OR (Cerebral Strokes):ab,ti,kw OR (Stroke, Cerebral):ab,ti,kw OR (Strokes, Cerebral):ab,ti,kw OR (Stroke, Acute):ab,ti,kw OR (Acute Stroke):ab,ti,kw OR (Acute Strokes):ab,ti,kw OR (Strokes, Acute):ab,ti,kw OR (Cerebrovascular Accident, Acute):ab,ti,kw OR (Acute Cerebrovascular Accident):ab,ti,kw OR (Acute Cerebrovascular Accidents):ab,ti,kw OR (Cerebrovascular Accidents, Acute):ab,ti,kw |
| #3 | #1 OR #2 |
| #4 | (Muscle Spasticity) |
| #5 | (Hemiplegia, Spastic):ab,ti,kw OR (Hemiplegias, Spastic):ab,ti,kw OR (Spastic Hemiplegia):ab,ti,kw OR (Spastic Hemiplegias):ab,ti,kw OR (Spasticity):ab,ti,kw OR (Spasticity, Muscle):ab,ti,kw OR (Spastic):ab,ti,kw OR (Clasp-Knife Spasticity):ab,ti,kw OR (Clasp Knife Spasticity):ab,ti,kw OR (Spasticity, Clasp-Knife):ab,ti,kw OR (Hypertonicity):ab,ti,kw OR (Muscle Hypertonia):ab,ti,kw OR (Hypertonia, Muscle):ab,ti,kw OR (Hypertonias, Muscle):ab,ti,kw OR (Muscle Hypertonias):ab,ti,kw OR (Muscle Tone Increased):ab,ti,kw OR (Increased, Muscle Tone):ab,ti,kw OR (Tone Increased, Muscle):ab,ti,kw OR (Muscular Hypertonicity):ab,ti,kw OR (Hypertonicities, Muscular):ab,ti,kw OR (Hypertonicity, Muscular):ab,ti,kw OR (Muscular Hypertonicities):ab,ti,kw OR (Hypermyotonia):ab,ti,kw OR (Hypermyotonias):ab,ti,kw |
| #6 | #4 OR #5 |
| #7 | (Upper Extremity) |
| #8 | (Extremities, Upper):ab,ti,kw OR (Upper Extremities):ab,ti,kw OR (Extremity, Upper):ab,ti,kw OR (Membrum superius):ab,ti,kw OR (Upper Limb):ab,ti,kw OR (Limbs, Upper):ab,ti,kw OR (Limb, Upper):ab,ti,kw OR (Upper Limbs):ab,ti,kw |
| #9 | #7 OR #8 |
| #10 | Acupuncture |
| #11 | (Electroacupuncture):ab,ti,kw OR (Manual needle):ab,ti,kw OR (body needle):ab,ti,kw OR (Needle):ab,ti,kw OR (acupuncture treatment):ab,ti,kw |
| #12 | #10 OR #11 |
| #13 | Massage |
| #14 | (Zone Therapy):ab,ti,kw OR (Therapies, Zone):ab,ti,kw OR (Zone Therapies):ab,ti,kw OR (Therapy, Zone):ab,ti,kw OR (Massage Therapy):ab,ti,kw OR (Massage Therapies):ab,ti,kw OR (Therapies, Massage):ab,ti,kw OR (Therapy, Massage):ab,ti,kw |
| #15 | #13 OR #14 |
| #16 | (Transcranial Magnetic Stimulation) |
| #17 | (Magnetic Stimulations, Transcranial):ab,ti,kw OR (Magnetic Stimulation, Transcranial):ab,ti,kw OR (Stimulations, Transcranial Magnetic):ab,ti,kw OR (Stimulation, Transcranial Magnetic):ab,ti,kw OR (Transcranial Magnetic Stimulations):ab,ti,kw OR (Transcranial Magnetic Stimulation, Paired Pulse):ab,ti,kw OR (Transcranial Magnetic Stimulation, Repetitive):ab,ti,kw OR (Transcranial Magnetic Stimulation, Single Pulse):ab,ti,kw |
| #18 | #16 OR #17 |
| #19 | (Low-Frequency Stimulation):ab,ti,kw OR (Low-Frequency Pulsed Eectrical Simulation):ab,ti,kw |
| #20 | #12 OR #15 OR #18 OR #19 |
| #21 | #3 AND #6 AND #9 AND #20 |

**The search strategy for China National Knowledge Infrastructure (CNKI) database**

| **No** | **Search History** |
| --- | --- |
| #1 | 主题：卒中 + 出血性卒中 + 缺血性卒中 + 中风 + 脑卒中 + 脑血管中风 + 脑血管意外 + 脑梗死 + 脑出血 + 脑中风（精确） |
| #2 | 主题：上肢痉挛偏瘫 + 上肢偏瘫, 痉挛 + 上肢痉挛性偏瘫 + 上肢痉挛型偏瘫 + 上肢硬瘫 + 上肢肌张力过强（精确） |
| #3 | 主题：针刺疗法 + 针灸疗法针刺 + 针灸 + 针法 + 体针+ 电针 + 低频电刺激 + 低频脉冲电刺激 + 经颅磁刺激+ 重复经颅磁刺激 + rTMS + 按摩 + 推拿 + 按摩推拿 + 推拿康复 +针刺穴位（精确） |
| #4 | (#1 AND #2 AND #3) |

**The search strategy for the Chinese Biological Medicine Database**

| **No** | **Search History** |
| --- | --- |
| #1 | "卒中"[全部字段: 智能] OR "中风"[全部字段: 智能] OR "脑血管意外"[不加权:扩展] OR "出血性卒中"[全部字段: 智能] OR "缺血性卒中"[全部字段: 智能] OR "脑卒中"[全部字段: 智能] OR "脑血管中风"[全部字段: 智能] OR "脑梗死"[全部字段: 智能] OR "脑出血"[全部字段: 智能] OR "脑中风"[全部字段: 智能] |
| #2 | "针刺疗法"[全部字段: 智能] OR "针灸疗法针刺"[全部字段: 智能] OR "针灸"[全部字段: 智能] OR "针灸"[全部字段: 智能] OR "针法"[全部字段: 智能] OR "体针"[全部字段: 智能] OR "电针"[全部字段: 智能] OR "低频电刺激"[全部字段: 智能] OR "低频脉冲电刺激"[全部字段: 智能] OR "经颅磁刺激"[全部字段: 智能] OR "重复经颅磁刺激"[全部字段: 智能] OR " rTMS "[全部字段: 智能] OR "按摩"[全部字段: 智能] OR "推拿"[全部字段: 智能] OR "按摩推拿"[全部字段: 智能] OR "推拿康复"[全部字段: 智能] OR "针刺穴位"[全部字段: 智能] |
| #3 | "上肢痉挛偏瘫"[全部字段: 智能] OR "上肢偏瘫, 痉挛"[全部字段: 智能] OR "上肢痉挛性偏瘫"[全部字段: 智能] OR "上肢痉挛型偏瘫"[全部字段: 智能] OR "上肢硬瘫"[全部字段: 智能] OR "上肢肌张力过强"[全部字段: 智能] |
| #4 | (#1 AND #2 AND #3) |

**The search strategy for the Chinese Science and Technology Periodical Database (VIP)**

| **No** | **Search History** |
| --- | --- |
| #1 | 题名或关键词=卒中 or 出血性卒中 or 缺血性卒中 or 中风 or 脑卒中 or 脑血管中风 or 脑血管意外 or 脑梗死 or 脑出血 or 脑中风 |
| #2 | 题名或关键词=针刺疗法 or 针灸疗法针刺 or 针灸 or针法or 体针 or 电针 or 低频电刺激 or 低频脉冲电刺激 or 经颅磁刺激 or 重复经颅磁刺激 or rTMS or 按摩 or 推拿 or 按摩推拿 or 推拿康复 or 针刺穴位 |
| #3 | 题名或关键词=上肢痉挛偏瘫 or 上肢偏瘫, 痉挛 or 上肢痉挛性偏瘫 or 上肢痉挛型偏瘫 or 上肢硬瘫 or 上肢肌张力过强 |
| #4 | (#1 AND #2 AND #3) |

**The search strategy for Wan Fang Database**

| **No** | **Search History** |
| --- | --- |
| #1 | 题名或关键词=卒中 or 出血性卒中 or 缺血性卒中 or 中风 or 脑卒中 or 脑血管中风 or 脑血管意外 or 脑梗死 or 脑出血 or 脑中风 |
| #2 | 题名或关键词=针刺疗法 or 针灸疗法针刺 or 针灸 or针法or 体针 or 电针 or 低频电刺激 or 低频脉冲电刺激 or 经颅磁刺激 or 重复经颅磁刺激 or rTMS or 按摩 or 推拿 or 按摩推拿 or 推拿康复 or 针刺穴位 |
| #3 | 题名或关键词=上肢痉挛偏瘫 or 上肢偏瘫, 痉挛 or 上肢痉挛性偏瘫 or 上肢痉挛型偏瘫 or 上肢硬瘫 or 上肢肌张力过强 |
| #4 | (#1 AND #2 AND #3) |
