# Supplementary material for: Comparison of the effects of different physical stimulation therapies on reducing upper limb spastic paralysis and motor dysfunction in stroke survivors after stroke: a network meta-analysis of randomized controlled trials
Source: Front Neurol. 2025 Apr 15;16:1554583. doi: 10.3389/fneur.2025.1554583 (PMC12037403; doi:10.3389/fneur.2025.1554583)
Supplement: Supplementary file 2 [file Data_Sheet_2.zip › supplementary Appendix S3.docx]

**Supplementary Appendix 3 Rating of overall quality using CONSORT guideline (N=49)**

| **Criteria** | **Description** | **Number of positive trials (n)** | **Percentage (%)** |
| --- | --- | --- | --- |
| **Title and abstract** | 1a Identification as a randomized trial in the title | 4 | 8.16 |
|  | 1b Structured summary of trial design, methods, results, and conclusions | 49 | 100 |
| **Introduction** | | | |
| Background and objectives | 2a Scientific background and explanation of rationale | 49 | 100 |
|  | 2b Specific objectives or hypotheses | 33 | 67.35 |
| **Methods** | | | |
| Trial design | 3a Description of trial design (such as parallel, factorial) including allocation ratio | 49 | 100 |
|  | 3b Important changes to methods after trial commencement (such as eligibility criteria), with reasons | 0 | 0 |
| Participants | 4a Eligibility criteria for participants | 47 | 95.92 |
|  | 4b Settings and locations where the data were collected | 46 | 93.88 |
| Interventions | 5 The interventions for each group with sufficient details to allow replication, including how and when they were actually administered | 46 | 93.88 |
| Outcomes | 6a Completely defined prespecified primary and secondary outcome measures, including how and when they were assessed | 49 | 100 |
|  | 6b Any changes to trial outcomes after the trial commenced, with reasons | 0 | 0 |
| Sample size | 7a How sample size was determined | 2 | 4.08 |
|  | 7b When applicable, explanation of any interim analyses and stopping guidelines | 0 | 0 |
| Randomization | 8a Method used to generate the random allocation sequence | 49 | 100 |
|  | 8b Type of randomization; details of any restriction (such as blocking and block size) | 33 | 67.35 |
| Allocation concealment | 9 Mechanism used to implement the random allocation sequence (such as sequentially numbered containers), describing any steps taken to conceal the sequence until interventions were assigned | 6 | 12.24 |
| Implementation | 10 Who generated the random allocation sequence, who enrolled participants, and who assigned participants to interventions | 9 | 18.37 |
| Blinding | 11a If done, who was blinded after assignment to interventions (for example, participants, care providers, those assessing outcomes) and how | 12 | 24.49 |
|  | 11b If relevant, description of the similarity of interventions | 1 | 2.04 |
| Statistical methods | 12a Statistical methods used to compare groups for primary and secondary outcomes | 49 | 100 |
|  | 12b Methods for additional analyses, such as subgroup analyses and adjusted analyses | 2 | 4.08 |
| **Results** | | | |
| Flow chart | 13a For each group, the numbers of participants who were randomly assigned, received intended treatment, and were analyzed for the primary outcome | 49 | 100 |
|  | 13b For each group, losses and exclusions after randomization, together with reasons | 3 | 6.12 |
| Recruitment | 14a Dates defining the periods of recruitment and follow-up | 41 | 83.67 |
|  | 14b Why the trial ended or was stopped | 0 | 0 |
| Baseline data | 15 A table showing baseline demographic and clinical characteristics for each group | 26 | 53.06 |
| Intent-to-treat analysis | 16 For each group, the number of participants (denominator) included in each analysis and whether the analysis was by originally assigned groups | 0 | 0 |
| Outcomes and estimation | 17a For each primary and secondary outcome, results for each group, and the estimated effect size and its precision (such as 95% confidence interval) | 49 | 100 |
|  | 17b For binary outcomes, presentation of both absolute and relative effect sizes is recommended | 23 | 46.94 |
| Ancillary analyses | 18 Results of any other analyses performed, including subgroup analyses and adjusted analyses, distinguishing pre-specified from exploratory | 0 | 0 |
| Harms | 19 All important harms or unintended effects in each group (for specific guidance see CONSORT for harms) | 16 | 32.65 |
| **Discussion** | | | |
| Limitations | 20 Trial limitations, addressing sources of potential bias, imprecision, and, if relevant, the multiplicity of analyses | 21 | 42.86 |
| Generalizability | 21 Generalizability (external validity, applicability) of the trial findings | 41 | 83.67 |
| Interpretation | 22 Interpretation consistent with results, balancing benefits and harms, and considering other relevant evidence | 42 | 85.71 |
| **Other information** | | | |
| Registration | 23 Registration number and name of trial registry | 5 | 10.20 |
| Protocol | 24 Where the full trial protocol can be accessed, if available | 5 | 10.20 |
| Funding | 25 Sources of funding and other support (such as supply of drugs), role of funders | 25 | 51.02 |
