# Supplementary material for: Comparison of the effects of different physical stimulation therapies on reducing upper limb spastic paralysis and motor dysfunction in stroke survivors after stroke: a network meta-analysis of randomized controlled trials
Source: Front Neurol. 2025 Apr 15;16:1554583. doi: 10.3389/fneur.2025.1554583 (PMC12037403; doi:10.3389/fneur.2025.1554583)
Supplement: Supplementary file 2 [file Data_Sheet_2.zip › Supplementary Figure.docx]

***Supplementary Figure***

***Comparison of the effects of different physical stimulation therapies on reducing upper limb spastic paralysis and motor dysfunction in stroke survivors after stroke: A network meta-analysis of randomized controlled trials***

**Mingtong Bian^1,2^, Fuyan Chen^1,2,3*^, Huizhen Su^3^, Zhiying Li^1,2^, Xiaowei Sun^1,2^, Yang Liu^1,2^,**

**Jinyuan Shi^1,2^, Shuo Liu^1,2^, Ru Rong^1,2^**

^1^Department of Acupuncture, First Teaching Hospital of Tianjin University of Traditional Chinese Medicine, Tianjin, China

^2^National Clinical Research Center for Chinese Medicine Acupuncture and Moxibustion, Tianjin, China

^3^Qinghai Provincial Hospital of Traditional Chinese Medicine,Qinghai,China

- **Figure S1** The result of the risk of bias assessment 3
- **Figure S2** The result of the inconsistency test 6
- **Figure S3** The results of Brooks-Gelman-Rubin diagnostic Plots 8
- **Figure S4** The results of trajectory and density Plots 11
- **Figure S5** The result of Sorting Probability Plot (SUCRA Plot), Cumulative Probability Plot for Ranking and Comparative Sorting Probability Plot 20

**Supplementary Fig. S1 The result of the risk of bias assessment.**

| **Study ID** | **D1** | **D2** | **D3** | **D4** | **D5** | **Overall** |
| --- | --- | --- | --- | --- | --- | --- |
| Ai YX 2023 | 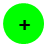 | 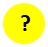 | 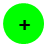 | 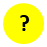 | 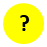 | 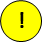 |
| Bao YH 2012 | 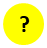 | 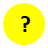 | 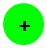 | 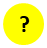 | 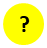 | 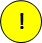 |
| Barros G 2014 | 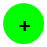 | 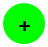 | 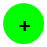 | 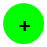 | 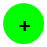 | 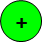 |
| Chen DY 2024 | 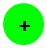 | 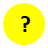 | 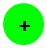 | 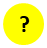 | 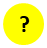 | 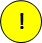 |
| Chen QF 2021 | 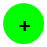 | 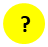 | 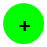 | 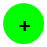 | 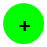 | 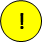 |
| Chen Y 2021 | 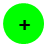 | 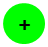 | 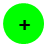 | 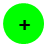 | 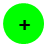 | 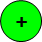 |
| Chen YJ 2019 | 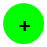 | 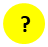 | 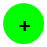 | 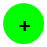 | 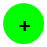 | 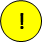 |
| Chu GX 2009 | 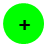 | 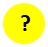 | 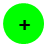 | 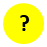 | 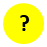 | 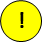 |
| Dang YS 2020 | 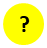 | 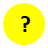 | 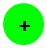 | 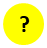 | 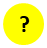 | 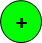 |
| Gu YL 2018 | 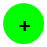 | 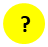 | 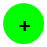 | 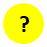 | 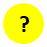 | 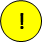 |
| Hao JB 2016 | 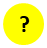 | 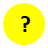 | 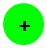 | 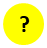 | 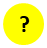 | 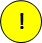 |
| Jiang YY 2023 | 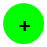 | 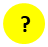 | 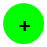 | 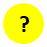 | 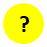 | 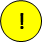 |
| Kuzu Ö 2021 | 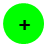 | 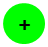 | 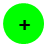 | 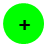 | 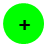 | 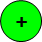 |
| Lei JF 2024 | 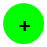 | 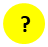 | 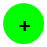 | 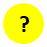 | 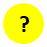 | 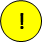 |
| Lei M 2012 | 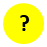 | 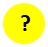 | 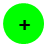 | 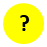 | 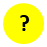 | 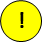 |
| Li B 2021 | 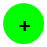 | 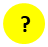 | 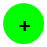 | 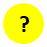 | 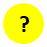 | 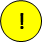 |
| Li BJ 2017 | 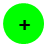 | 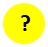 | 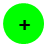 | 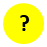 |  |  |
| Li D 2021 |  |  |  |  |  |  |
| Li ZW 2022 |  |  |  |  |  |  |
| Lin FY 2018 |  |  |  |  |  |  |
| Liu HJ 2023 |  |  |  |  |  |  |
| Liu QQ 2021 |  |  |  |  |  |  |
| Liu SD 2023 |  |  |  |  |  |  |
| Liu SH 2019 |  |  |  |  |  |  |
| Liu Y 2018 |  |  |  |  |  |  |
| Ma AF 2022 |  |  |  |  |  |  |
| Ma JY 2020 |  |  |  |  |  |  |
| Motamed V 2014 |  |  |  |  |  |  |
| Ni HH 2012 |  |  |  |  |  |  |
| Qin Y 2023 |  |  |  |  |  |  |
| Shi J 2019 |  |  |  |  |  |  |
| Sun X 2023 |  |  |  |  |  |  |
| Sun YZ 2013 |  |  |  |  |  |  |
| Tong JY 2022 |  |  |  |  |  |  |
| Wang CP 2014 |  |  |  |  |  |  |
| Wang J 2018 |  |  |  |  |  |  |
| Wei CB 2021 |  |  |  |  |  |  |
| Wen DG 2020 |  |  |  |  |  |  |
| Xie WX 2023 |  |  |  |  |  |  |
| Xu SF 2016 |  |  |  |  |  |  |
| Xu YL 2010 |  |  |  |  |  |  |
| Yang NY 2017 |  |  |  |  |  |  |
| Yang X 2021 |  |  |  |  |  |  |
| Zhang L 2015 |  |  |  |  |  |  |
| Zhang QF 2021 |  |  |  |  |  |  |
| Zhang X 2021 |  |  |  |  |  |  |
| Zhao J 2021 |  |  |  |  |  |  |
| Zhao JY 2021 |  |  |  |  |  |  |
| Zhou P 2019 |  |  |  |  |  |  |
|  |  | | |  |  |  |

|  | Low risk |
| --- | --- |
|  | Some concerns |
|  | High risk |
| D1 | Randomisation process |
| D2 | Deviations from the intended interventions |
| D3 | Missing outcome data |
| D4 | Measurement of the outcome |
| D5 | Selection of the reported result |

**Supplementary Fig. S2 The result of the inconsistency test.**

**FMA-UE**

**MBI**

A: physical rehabilitation; B: Body acupuncture; C: Electro-acupuncture; D: Massage; E: Proprioceptive Neuromuscular Facilitation; F: Body acupuncture plus extracorporeal shock wave treatment; G: Body acupuncture plus proprioceptive neuromuscular facilitation; H: Body acupuncture plus massage; I: Electro-acupuncture plus repetitive transcranial magnetic stimulation; J: Physical rehabilitation plus continuous theta burst stimulation; K: Physical rehabilitation plus intermittent theta burst stimulation; L: Physical rehabilitation plus repetitive transcranial magnetic stimulation; M: Physical rehabilitation plus extracorporeal shock wave treatment; N: Physical rehabilitation plus electro-acupuncture; O: Physical rehabilitation plus body acupuncture; P: Physical rehabilitation plus massage; Q: Physical rehabilitation plus repetitive transcranial magnetic stimulation plus continuous theta burst stimulation; R: Physical rehabilitation plus repetitive transcranial magnetic stimulation plus intermittent theta burst stimulation; S: Physical rehabilitation plus repetitive transcranial magnetic stimulation plus body acupuncture; T: Physical rehabilitation plus extracorporeal shock wave treatment plus body acupuncture; U: Physical rehabilitation plus repetitive transcranial magnetic stimulation plus electro-acupuncture; FMA-UE: The Fugl-Meyer Assessment-Upper Extremity scale; MBI: The Modified Barthel Index scale.

**Supplementary Fig. S3 The result of Brooks-Gelman-Rubin diagnostic Plots.**

**FMA-UE**

**MBI**

A: Physical rehabilitation; B: Body acupuncture; C: Electro-acupuncture; D: Massage; E: Proprioceptive Neuromuscular Facilitation; F: Body acupuncture plus extracorporeal shock wave treatment; G: Body acupuncture plus proprioceptive neuromuscular facilitation; H: Body acupuncture plus massage; I: Electro-acupuncture plus repetitive transcranial magnetic stimulation; J: Physical rehabilitation plus continuous theta burst stimulation; K: Physical rehabilitation plus intermittent theta burst stimulation; L: Physical rehabilitation plus repetitive transcranial magnetic stimulation; M: Physical rehabilitation plus extracorporeal shock wave treatment; N: Physical rehabilitation plus electro-acupuncture; O: Physical rehabilitation plus body acupuncture; P: Physical rehabilitation plus massage; Q: Physical rehabilitation plus repetitive transcranial magnetic stimulation plus continuous theta burst stimulation; R: Physical rehabilitation plus repetitive transcranial magnetic stimulation plus intermittent theta burst stimulation; S: Physical rehabilitation plus repetitive transcranial magnetic stimulation plus body acupuncture; T: Physical rehabilitation plus extracorporeal shock wave treatment plus body acupuncture; U: Physical rehabilitation plus repetitive transcranial magnetic stimulation plus electro-acupuncture; FMA-UE: The Fugl-Meyer Assessment-Upper Extremity scale; MBI: The Modified Barthel Index scale.

**Supplementary Fig. S4 The result of trajectory and density Plots.**

**FMA-UE**

**MBI**

A: Physical rehabilitation; B: Body acupuncture; C: Electro-acupuncture; D: Massage; E: Proprioceptive Neuromuscular Facilitation; F: Body acupuncture plus extracorporeal shock wave treatment; G: Body acupuncture plus proprioceptive neuromuscular facilitation; H: Body acupuncture plus massage; I: Electro-acupuncture plus repetitive transcranial magnetic stimulation; J: Physical rehabilitation plus continuous theta burst stimulation; K: Physical rehabilitation plus intermittent theta burst stimulation; L: Physical rehabilitation plus repetitive transcranial magnetic stimulation; M: Physical rehabilitation plus extracorporeal shock wave treatment; N: Physical rehabilitation plus electro-acupuncture; O: Physical rehabilitation plus body acupuncture; P: Physical rehabilitation plus massage; Q: Physical rehabilitation plus repetitive transcranial magnetic stimulation plus continuous theta burst stimulation; R: Physical rehabilitation plus repetitive transcranial magnetic stimulation plus intermittent theta burst stimulation; S: Physical rehabilitation plus repetitive transcranial magnetic stimulation plus body acupuncture; T: Physical rehabilitation plus extracorporeal shock wave treatment plus body acupuncture; U: Physical rehabilitation plus repetitive transcranial magnetic stimulation plus electro-acupuncture; FMA-UE: The Fugl-Meyer Assessment-Upper Extremity scale; MBI: The Modified Barthel Index scale.

**Supplementary Fig. S5 The result of Sorting Probability Plot (SUCRA Plot), Cumulative Probability Plot for Ranking and** **Comparative Sorting Probability Plot.**

**FMA-UE**

**Figure S5 A1. Sorting Probability Plot (SUCRA Plot)**

**Figure S5 A2. Cumulative Probability Plot for Ranking**

**Figure S5 A3. Comparative Sorting Probability Plot**

**MBI**

**Figure S5 B1. Sorting Probability Plot (SUCRA Plot)**

**Figure S5 B2. Cumulative Probability Plot for Ranking**

**Figure S5 B3. Comparative Sorting Probability Plot**

A: Physical rehabilitation; B: Body acupuncture; C: Electro-acupuncture; D: Massage; E: Proprioceptive Neuromuscular Facilitation; F: Body acupuncture plus extracorporeal shock wave treatment; G: Body acupuncture plus proprioceptive neuromuscular facilitation; H: Body acupuncture plus massage; I: Electro-acupuncture plus repetitive transcranial magnetic stimulation; J: Physical rehabilitation plus continuous theta burst stimulation; K: Physical rehabilitation plus intermittent theta burst stimulation; L: Physical rehabilitation plus repetitive transcranial magnetic stimulation; M: Physical rehabilitation plus extracorporeal shock wave treatment; N: Physical rehabilitation plus electro-acupuncture; O: Physical rehabilitation plus body acupuncture; P: Physical rehabilitation plus massage; Q: Physical rehabilitation plus repetitive transcranial magnetic stimulation plus continuous theta burst stimulation; R: Physical rehabilitation plus repetitive transcranial magnetic stimulation plus intermittent theta burst stimulation; S: Physical rehabilitation plus repetitive transcranial magnetic stimulation plus body acupuncture; T: Physical rehabilitation plus extracorporeal shock wave treatment plus body acupuncture; U: Physical rehabilitation plus repetitive transcranial magnetic stimulation plus electro-acupuncture; FMA-UE: The Fugl-Meyer Assessment-Upper Extremity scale; MBI: The Modified Barthel Index scale.
